# Evaluating Memory B Cell Cross-Reactivity Between Ancestral and Future SARS-CoV-2 Variants—Evidence for Original Antigenic Sin

**DOI:** 10.3390/vaccines14070604

**Published:** 2026-07-09

**Authors:** Lingling Yao, Zoltán Megyesi, Paul V. Lehmann, Greg A. Kirchenbaum

**Affiliations:** 1 Research and Development, Cellular Technology Ltd. (CTL), Shaker Heights, OH 44122, USA; lingling.yao@immunospot.com (L.Y.); paul.lehmann@immunospot.com (P.V.L.); 2 CTL Software Ltd., Vágó str. 2, H-6000 Kecskemét, Hungary; zoltan.megyesi@immunospot.com

**Keywords:** ELISPOT, immunological memory, antigenic imprinting, antigenic seniority, high-content analysis, vaccine, immune monitoring

## Abstract

**Background**: Despite the circulation of evolutionarily related cold-causing coronaviruses (CCCs) in the pre-COVID era, most individuals lacked pre-existing serum IgG and/or class-switched memory B cell (B_mem_) reactivity for the SARS-CoV-2 Spike (S) glycoprotein expressed by the ancestral Wuhan-Hu-1 (WH1) strain. Subsequent priming of the immune system through natural infection or prophylactic COVID-19 mRNA vaccination successfully generated robust B_mem_ responses against the WH1-S antigen, along with eliciting cross-reactivity for the future Omicron (BA.1) variant responsible for breakthrough infections (BTIs). However, to what extent immunological imprinting of B_mem_ towards the WH1-S antigen detrimentally constrains the elicitation of variant-specific antibody responses following subsequent booster vaccinations or BTIs—a phenomena referred to as “original antigenic sin”—remains an unresolved and open question. **Methods**: Using ImmunoSpot^®^, we evaluated peripheral blood mononuclear cells (PBMCs) from defined human cohorts for IgG^+^ ASC reactivity against Spike proteins representing CCCs and SARS-CoV-2. Additionally, we developed a novel dual-label inverted FluoroSpot assay to distinguish between strain-specific and cross-reactive IgG^+^ ASCs recognizing epitopes in the receptor binding domain (RBD) of SARS-CoV-2 Omicron variants. **Results**: Our data demonstrate a lack of appreciable back-boosting of IgG^+^ B_mem_ recognizing structurally conserved epitopes shared between CCCs and SARS-CoV-2. Moreover, we found evidence for immunological imprinting and the preferential expansion of B_mem_ recognizing cross-reactive epitopes in the RBD following BTI. Nevertheless, Omicron strain-specific B_mem_ were detected in PBMC donors collected in 2025. **Conclusions**: Our novel inverted dual-label FluoroSpot methodology evidenced preferential expansion of cross-reactive B_mem_ following breakthrough SARS-CoV-2 infection and supports the influence of original antigenic sin shaping the recall response. Moreover, the inverted dual-label assay provides a highly flexible and easily implementable technique for distinguishing between strain-specific and cross-reactive B cell responses and has broad applications in translational vaccine research against pathogens that undergo antigenic drift.

## 1. Introduction

Severe acute respiratory syndrome coronavirus 2 (SARS-CoV-2) was first identified in late 2019 in Wuhan, China [1,2]. The United States confirmed its first case shortly after on 20 January 2020 [3]. While evolutionarily related cold-causing coronaviruses (CCCs) have long circulated in the human population [4,5,6,7,8,9,10], the global population lacked protective immunity at the onset of the coronavirus disease 2019 (COVID-19) pandemic. Most individuals lacked baseline serum IgG and/or class-switched memory B cell reactivity against the Spike (S) glycoprotein expressed by the novel SARS-CoV-2 pathogen [11,12,13,14,15,16,17]. Consequently, widespread global infections occurred [18] and necessitated the rapid development and deployment of COVID-19 vaccines [19].

Messenger RNA (mRNA) vaccines developed by Pfizer/BioNTech and Moderna encoded the S protein of the ancestral Wuhan-Hu-1 (WH1) strain. These vaccines potently elicited neutralizing antibody reactivity in both naïve and convalescent subjects [20,21,22,23]. Furthermore, by triggering long-lasting germinal center reactions in draining lymph nodes [24], COVID-19 mRNA vaccines generated robust memory B cell (B_mem_) responses. These B_mem_ continued to increase in frequency and underwent further affinity maturation over the course of several months [24,25,26].

Even before the emergence of the BA.1 (Omicron) variant, two mRNA doses elicited cross-reactive serum IgG and B_mem_ responses against this future SARS-CoV-2 strain [27,28]. However, to counteract waning immunity [29,30,31], a third vaccine dose was recommended in late 2021. Booster vaccines successfully replenished protective serum antibody titers and enhanced both neutralizing activity and the frequency of B_mem_ recognizing the Omicron lineage [32,33,34,35,36]. Nevertheless, despite this latent cross-reactivity, the Omicron lineage quickly became the dominant global strain in early 2022 [37].

Compared to the ancestral WH1 strain, the newly emerged Omicron variant harbored more than 30 mutations in its S protein [37]. Consequently, these mutations—many of which are located within the receptor binding domain (RBD)—reduced the protective efficacy of therapeutic monoclonal antibodies (mAbs), as well as the immunity acquired through prior infection or vaccination [38,39,40]. Widespread breakthrough infections (BTIs) further motivated the reformulation of COVID-19 vaccines to include Omicron variant strains [41]. However, evaluation of immunity elicited by updated COVID-19 mRNA vaccines revealed that imprinting fundamentally shaped the recall response. Specifically, serum absorption and/or antigen probe-based flow cytometry studies demonstrated that these boosters preferentially expanded pre-existing, cross-reactive B_mem_ populations rather than eliciting de novo, strain-specific responses against Omicron [42,43,44]. Overcoming this phenomenon, historically termed “original antigenic sin”, remains a paramount challenge hindering the generation of vaccines capable of eliciting broadly protective immunity against emerging SARS-CoV-2 variants [45].

Direct assessments of antigen-specific B_mem_ provide invaluable insights into their precursor frequency and the specificity of recall responses. Unlike serum antibody reactivity measurements, characterization of B_mem_ can predict the specificity of secondary immune responses following antigen re-exposure. While antigen probe-based flow cytometry approaches can be technically challenging [46,47], the ImmunoSpot assay methodology provides an alternative strategy for studying B_mem_ at single-cell resolution [48]. However, in the absence of a recent antigen encounter, B_mem_ are resting lymphocytes that do not spontaneously secrete antibodies. Consequently, an in vitro polyclonal stimulation culture is required to convert B_mem_ into antibody-secreting cells (ASCs) that can be detected based on the generation of antibody-derived secretory footprints.

Having previously detected B_mem_ cross-reactivity for the BA.1 Omicron RBD following either a single infection or the initial two-dose vaccination with ancestral (WH1) Spike [28], here we sought to extend these observations. In the present study, we utilize a novel dual-label inverted ImmunoSpot^®^ assay to determine whether these variant RBD-reactive B_mem_ are strictly strain-specific, or if they target structurally conserved epitopes shared between the ancestral (WH1) and Omicron (BA.1) strains. Furthermore, we clarify whether BTIs successfully elicit de novo, variant-specific B cell responses or if they primarily back-boost pre-existing, cross-reactive B_mem_. Lastly, we evaluate PBMC samples collected in 2025 to ascertain whether repetitive exposure to variant S protein—through repeated infection or vaccination—can eventually overcome immune imprinting to generate dedicated, Omicron-specific B_mem_.

## 2. Materials and Methods

### 2.1. Human Subjects

Peripheral blood mononuclear cell (PBMC) samples originating from four defined cohorts were characterized in this study. The first cohort consisted of pre-COVID-19 era samples (*n* = 22) that were collected prior to November 2019. The second cohort consisted of convalescent donors (*n* = 8) who recovered from polymerase chain reaction (PCR)-verified SARS-CoV-2 infections occurring early in the pandemic; samples were collected prior to January 2021, before COVID-19 vaccines became widely available in the United States. The third cohort consisted of donors (*n* = 10) with PCR-verified breakthrough infections (BTIs) occurring between December 2021 and January 2022. Samples in this cohort were collected between January and February 2022, coinciding with the timeframe in which Omicron variants were responsible for most SARS-CoV-2 infections [37,49]. Lastly, the fourth cohort consisted of post-COVID era donors (*n* = 45) whose samples were collected between February 2023 and October 2025.

Samples from cohort 1 (pre-COVID), cohort 2 (convalescent), and cohort 4 (post-COVID) were collected at FDA-registered collection centers from IRB-consented healthy human donors via leukapheresis. These samples were then sold to CTL, identifying donors by code only while concealing the subjects’ identities. Additionally, blood samples from cohort 3 (BTI) were collected internally at CTL under an Advarra-approved IRB #Pro00043178 (CTL contract laboratory study number GL20-16 entitled COVID-19 Immune Response Evaluation). All PBMC samples were isolated and cryopreserved according to previously described protocols [50,51] and were stored in liquid nitrogen until testing. Details of all human donors included in this manuscript, including demographics, collection dates, and SARS-CoV-2 infection/COVID-19 vaccination history (if known), are provided in Supplemental Table S1.

### 2.2. Polyclonal B Cell Stimulation

Detailed methods of the thawing, washing and counting of PBMCs have been previously described [51,52]. Freshly thawed PBMC samples were resuspended in complete medium (CM) containing RPMI 1640 (Alkali Scientific, Fort Lauderdale, FL, USA) supplemented with 10% fetal bovine serum (Gemini Bioproducts, West Sacramento, CA, USA), 100 U/mL penicillin, 100 U/mL streptomycin, 2 mM L-glutamine, 1 mM sodium pyruvate, 8 mM HEPES (all from Life Technologies, Grand Island, NY, USA), and 50 µM β-mercaptoethanol (Sigma-Aldrich, St. Louis, MO, USA). PBMCs were then stimulated with Human B-Poly-S (CTL) containing TLR7/8 agonist R848 (1 µg/mL final concentration) and recombinant human IL-2 (10 ng/mL final concentration) [53,54] at a density of 0.5–2 × 10^6^ cells/mL in 25 cm^2^ or 75 cm^2^ tissue culture flasks (Corning, Sigma-Aldrich) and incubated at 37 °C, 5% CO_2_ for 5 days to promote the terminal differentiation of resting B cells into antibody-secreting cells (ASCs) prior to evaluation in ImmunoSpot^®^ assays.

### 2.3. Recombinant Proteins

Full-length, trimeric SARS-CoV-2 Spike (S) protein representing the ancestral Wuhan-Hu-1 strain [55], denoted as WH1-S (FL), and a truncated version encoding only the receptor binding domain (RBD) with a genetically encoded His affinity tag [56], denoted as WH1-S (RBD)-His, were acquired from the Center for Vaccines and Immunology (CVI) (University of Georgia, Athens, GA, USA). Full-length, trimeric hemagglutinin protein representing the A/California/04/2009 H1N1 influenza A virus strain, denoted as CA/09 rHA, was also obtained from the CVI and has been described previously [57]. Full-length S protein representing the human cold-causing coronavirus (CCC) NL63 strain (Cat #100788), denoted as NL63-S (FL), was purchased from BPS Bioscience (San Diego, CA, USA). RBD proteins representing CCC strains 229E (Cat #10612-CV), NL63 (Cat #10605-CV), and HKU1 (Cat #10600-CV) were purchased from R&D Systems (Minneapolis, MN, USA). RBD protein representing the ancestral WH1 strain of SARS-CoV-2 with a genetically encoded FLAG affinity tag, denoted as WH1-S (RBD)-FLAG, was also purchased from R&D Systems (Cat #10689-CV). His-tagged RBD protein representing the SARS-CoV-2 BA.1 Omicron variant (Cat #Spike-678V) was purchased from Creative Biomart (Shirley, NY, USA). His-tagged RBD proteins representing the SARS-CoV-2 JN.1 (Cat #SPD-C5249) or XFG (Cat #SPD-S52Hf) variants were purchased from ACRO Biosystems (Newark, DE, USA). Notably, all recombinant proteins used in this study, except the WH1-S (RBD)-FLAG, possessed a genetically encoded His affinity tag.

### 2.4. B Cell ImmunoSpot^®^ Assays

#### 2.4.1. Antigen-Specific ELISPOT Assays with Affinity Capture Coating

For detection of antigen-specific IgG^+^ ASCs in single-color ELISPOT assays using the affinity capture coating method [58], PVDF membrane-bottom 96-well ImmunoSpot^®^ assay plates (CTL) were pre-conditioned with 70% (*v*/*v*) EtOH (15 µL/well) followed by two washes with phosphate-buffered saline (PBS) (150 µL/well) prior to coating with purified anti-His antibody at 10 µg/mL in Diluent A (provided in CTL’s affinity coating kits) overnight at 4 °C. The following day, assay plates were washed once with 150 µL/well of PBS and then blocked with 150 µL/well of CM for at least 1 h at room temperature (RT) prior to coating overnight at 4 °C with His-tag labeled WH1-S (FL) or CA/09 rHA protein at 10 µg/mL in Diluent A. Prior to use, assay plates were washed once with 150 µL/well of PBS. Immediately prior to plating cells, assay plates were decanted, and 100 µL of pre-warmed CM was added to each well.

PBMCs were harvested after 5 days of polyclonal stimulation [28,53] and washed twice with PBS prior to counting using CTL’s Live/Dead Cell Counting Suite on an S6 Flex M2 Analyzer (CTL). After centrifugation, cell pellets were resuspended at 5 × 10^6^ live cells/mL in CM prior to plating 100 µL (5 × 10^5^ live cells) into three replicate wells coated with WH1-S (FL) protein. Alternatively, donor PBMCs were tested for IgG^+^ ASC reactivity for CA/09 rHA using a two-fold serial dilution approach starting at 5 × 10^5^ live cells per well, with three additional cell inputs in singlet. To avoid damage to the assay membrane, PBMCs were serially diluted in round-bottom 96-well tissue culture plates (Corning, Sigma-Aldrich) and then subsequently transferred into assay plates as previously described [28]. Following plating, assay plates were incubated for 4–6 h at 37 °C, 5% CO_2_. Plate-bound spot-forming units (SFUs), each representing the secretory footprint of a single IgG^+^ ASC, were visualized using IgG-specific detection reagents included in the human IgG Single-Color Enzymatic ImmunoSpot^®^ kit (CTL, Shaker Heights, OH, USA) according to the manufacturer’s instructions.

#### 2.4.2. Pan IgG ELISPOT Assays

To verify the functionality of polyclonally stimulated PBMC samples, pan (total) IgG assays were performed using reagents included in Single-Color Enzymatic ImmunoSpot^®^ kits (CTL) according to the manufacturer’s instructions. Specifically, donor PBMCs were diluted to 2 × 10^5^ live cells/mL and then serially diluted two-fold in round-bottom 96-well tissue culture plates prior to transfer into assay plates as previously described [28]. Assay plates were then incubated for 4–6 h at 37 °C, 5% CO_2_. Plate-bound SFUs, each representing the secretory footprint of a single IgG^+^ ASC irrespective of antigen specificity, were visualized as described above.

#### 2.4.3. Antigen-Specific FluoroSpot Assays with Affinity Capture Coating

FluoroSpot-based detection of antigen-specific IgG^+^ ASCs using affinity capture coating was performed using similar methods as previously described [28,58]. In brief, low autofluorescence assay plates (CTL) were pre-wet with 70% (*v*/*v*) EtOH (15 µL/well) followed by two washes with PBS (150 µL/well). Assay wells were then coated with purified anti-His antibody at 10 µg/mL in Diluent A (provided in CTL’s affinity coating kits) overnight at 4 °C. The following day, assay plates were washed once with 150 µL/well of PBS and then blocked with 150 µL/well of CM for at least 1 h at RT. Plates were then decanted and coated overnight at 4 °C with His-tag labeled WH1-S (FL) or NL63-S (FL) protein at 10 µg/mL in Diluent A. The following day, assay plates were washed once more with 150 µL/well of PBS. Prior to plating cells, assay plates were decanted and 100 µL of pre-warmed CM was added to each well.

Polyclonally stimulated PBMC samples were harvested, washed, and counted as described above. After centrifugation, cell pellets were resuspended at 5 × 10^6^ live cells/mL in CM prior to plating 100 µL (5 × 10^5^ live cells) into four replicate wells coated with NL63-S protein. Alternatively, PBMC samples were diluted to 1 × 10^6^ live cells/mL in CM and then serially diluted two-fold in round-bottom 96-well tissue culture plates prior to transfer into WH1-S (FL)-coated assay wells as previously described [28]. Assay plates were then incubated overnight (~16 h) at 37 °C, 5% CO_2_. Plate-bound SFUs, each representing the secretory footprint of a single IgG^+^ ASC, were visualized using IgG-specific detection reagents included in the multiplexed human IgA/IgG/IgM ImmunoSpot^®^ kit (CTL) according to the manufacturer’s instructions. Additionally, the functionality of polyclonally stimulated PBMCs was evaluated in pan (total) IgG assays performed in parallel using reagents included in the human IgA/IgG/IgM ImmunoSpot^®^ kit (CTL) according to the manufacturer’s instructions using methods similar to those described above.

#### 2.4.4. Single-Color Inverted ImmunoSpot^®^ Assays

To evaluate IgG^+^ ASC reactivity for RBD probes representing CCC (229E, NL63 or HKU1) or SARS-CoV-2 (WH1 or BA.1), polyclonally stimulated PBMC samples were tested in Single-Color IgG-specific inverted ImmunoSpot^®^ assays using His-tagged RBD proteins as described previously [28]. In brief, low autofluorescence FluoroSpot plates (CTL) were pre-conditioned with 70% (*v*/*v*) EtOH (15 µL/well) followed by two washes with PBS (150 µL/well) prior to coating with purified anti-human IgG Fc capture antibody at 15 µg/mL in Diluent A (provided in CTL’s inverted ImmunoSpot^®^ kits) overnight at 4 °C. The following day, assay plates were washed and blocked as described above. Polyclonally stimulated PBMCs were harvested, washed, and counted as described above. PBMCs were then plated at 1 × 10^5^ live cells per well into three wells for the detection of IgG^+^ ASC reactivity against RBD probes representing different CCC (each tested individually in single wells), or were tested using a two-fold serial dilution approach (in singlet) starting at 1 × 10^5^ live cells per well to evaluate IgG^+^ ASC reactivity for RBD probes representing either the ancestral (WH1) or Omicron variant (BA.1) SARS-CoV-2 strains. Assay plates were then incubated overnight (~16 h) at 37 °C, 5% CO_2_. Plate-bound SFUs were subsequently visualized using His-tagged RBD proteins at 100 ng/mL in Diluent B (provided in CTL’s inverted ImmunoSpot^®^ kits) for 2 h at RT. After decanting and washing, anti-His detection antibody conjugated to Alexa Fluor^®^ 488 (provided in CTL’s His inverted ImmunoSpot^®^ kit) was used according to the manufacturer’s instructions to visualize RBD-reactive IgG^+^ SFUs.

Alternatively, assay plates were incubated for 5 h at 37 °C, 5% CO_2_ to compare the sensitivity of enzymatic- and fluorescence-based assays using His-tagged or FLAG-tagged WH1-S RBD probes. Plate-bound SFUs were subsequently visualized using anti-His or anti-FLAG detection reagents (provided in CTL’s inverted ImmunoSpot^®^ kits) according to the manufacturer’s instructions.

#### 2.4.5. Dual-Label Inverted ImmunoSpot^®^ Assays

To distinguish between strain-specific and cross-reactive IgG^+^ ASCs, polyclonally stimulated PBMCs were plated into inverted ImmunoSpot^®^ assays at donor-specific cell inputs not exceeding 5 × 10^4^ live cells per well to achieve ≤50 WH1-S (RBD)-specific SFUs in replicate wells. Assay plates were then incubated for 5 h at 37 °C, 5% CO_2_. His-tagged RBD probe representing either the ancestral (WH1) or a variant (BA.1, JN.1 or XFG) SARS-CoV-2 strain was combined with WH1-S (RBD)-FLAG probe (each at 50 ng/mL in Diluent B), added into designated wells (*n* = 24), and plates were incubated overnight at 4 °C. Plate-bound SFUs were subsequently visualized using anti-His or anti-FLAG detection reagents (provided in CTL’s dual-label inverted ImmunoSpot^®^ kits) according to the manufacturer’s instructions.

#### 2.4.6. Image Acquisition and SFU Counting

ELISPOT or FluoroSpot plates were air-dried prior to scanning on an S6 Flex M2 Analyzer using ImmunoSpot^®^ software (Version 7.0.38.17) (CTL). Quantification of SFUs in antigen-specific or pan (total) IgG ELISPOT assays was performed using ImmunoSpot^®^ Single-color Studio software (Version 1.7.37.0) and B cell IntelliCount^™^ algorithms [59]. Quantification of SFUs in FluoroSpot assays was performed using either the Fluoro-X^™^ suite of ImmunoSpot^®^ software (Version 7.0.38.17) or ImmunoSpot^®^ Studio software (Version 1.0.8.0) and the Basic Count mode (CTL). Dual-labeled SFUs were additionally identified using a previously described center of mass distance algorithm [60]. Individual ELISPOT or FluoroSpot well images were quality controlled to remove artifacts and improve the accuracy of counts as needed. Only SFU counts within the linear titration range of the ImmunoSpot^®^ assay, or SFU counts from the highest cell input tested, were considered for frequency calculations. For the high-content analysis of secretory footprint morphology in dual-label inverted assays, the area of the SFU (denoted as size) in the individual detection channels were measured independently. Moreover, replicate wells were merged into a single flow cytometry standard (FCS) file using ImmunoSpot^®^ Studio software (Version 1.0.8.0) and subsequently analyzed using FlowJo^™^ v10.9 software (BD Life Sciences). Since ImmunoSpot^®^ B cell kits, analyzers, and software proprietary to CTL were used in this study, we refer to the collective methodology as ImmunoSpot^®^.

### 2.5. Statistical Methods

Significant differences in the frequency of single-positive (SP) and/or double-positive (DP) SFUs between donor cohorts in dual-label inverted ImmunoSpot^®^ assays using different RBD probe combinations were determined using Welch’s analysis of variance (ANOVA) tests with Dunnett’s T3 post hoc correction for multiple comparisons (GraphPad Prism Version 11.0.0, San Diego, CA, USA). Similarly, Welch’s ANOVA with Dunnett’s T3 correction was performed to compare the frequency of DP SFUs that were larger in the red or green detection channels, or those equal in size, between the donor cohorts. For both analyses, it was assumed that the data followed a normal (Gaussian) distribution.

## 3. Results

### 3.1. Lack of Substantial Epitope Conservation Between Cold-Causing Coronaviruses and SARS-CoV-2 Spike Proteins

Cold-causing coronavirus (CCC) strains, such as alphacoronaviruses 229E and NL63 or betacoronaviruses HKU1 and OC43, were already known to circulate in the human population prior to the introduction of SARS-CoV-2 [4,5,6,7,8,9,10]. While evolutionarily related, the Spike proteins expressed by endemic CCCs and SARS-CoV-2 possess very low sequence identity [61]. Nevertheless, it is plausible that memory B cells (B_mem_) primed by prior infections with CCCs could cross-react with epitopes within the SARS-CoV-2 Spike owing to shared structural homology, providing some level of protection following infection. Moreover, such pre-existing B_mem_ could shape the ensuing B cell response engaged after SARS-CoV-2 infection or COVID-19 vaccination.

**Figure 1:**
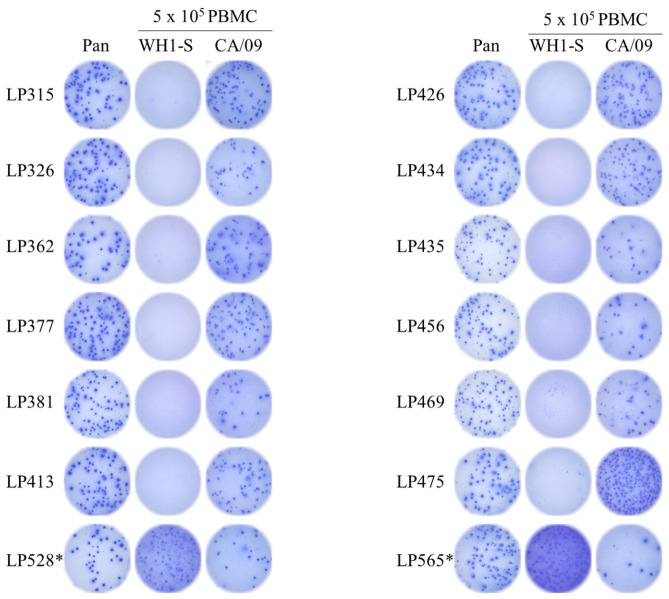
Pre-COVID era donors lack B_mem_-derived IgG^+^ ASC cross-reactivity for SARS-CoV-2 Spike (WH1-S). Representative well images depicting ImmunoSpot^®^ assay results from polyclonally stimulated PBMCs (5 × 10^5^ cells/well) of pre-COVID era donors tested in WH1-S-coated wells. LP528 and LP565, denoted by asterisks, are convalescent donors with PCR-verified SARS-CoV-2 exposure that served as positive controls for WH1-S-specific IgG^+^ ASC detection. (refer to Supplementary Table S1). Functionality of the cryopreserved PBMCs following polyclonal stimulation with B-Poly-S (R848+rIL-2) was verified in pan (total) IgG^+^ ImmunoSpot assays (see Supplementary Table S2 for calculated pan IgG^+^ SFU per 1.5 × 10^6^ PBMCs). Parallel control wells depict positive ImmunoSpot^®^ test results obtained when 5 × 10^5^ PBMCs were plated into wells coated with recombinant hemagglutinin protein representing an H1N1 influenza strain (A/California/04/2009, CA/09).

We first sought to address whether structural homology between CCCs and SARS-CoV-2 Spike proteins could shape the immune response by determining if B_mem_ recognizing the ancestral SARS-CoV-2 Spike protein, denoted as WH1-S (FL) hereafter, could be detected in donors’ PBMCs collected in the pre-COVID era (before November 2019). PBMC samples collected from convalescent donors with PCR-verified SARS-CoV-2 infection served as positive controls. Single-color ELISPOT assays were performed (illustrated in Supplementary Figure S1A) and donors (*n* = 22) were tested at 5 × 10^5^ PBMCs per well in triplicate to improve the detection limit of the assay [62]. Raw data illustrating the results obtained when testing PBMCs from twelve pre-COVID era donors are shown in Figure 1.

Whereas B_mem_-derived IgG^+^ ASC reactivity for WH1-S (FL) was detectable in both positive control samples—being so abundant that individual SFUs could not be reliably enumerated at 5 × 10^5^ PBMCs per well—no WH1-S (FL)-reactive IgG^+^ ASCs were detected when testing pre-COVID era samples despite screening 1.5 × 10^6^ PBMCs in aggregate (Supplementary Table S2). In stark contrast to this experimental outcome, WH1-S (FL)-reactive IgG^+^ ASCs were detectable in donor PBMCs collected after 2023, albeit at variable frequencies (Supplementary Table S3). Importantly, the lack of detectable WH1-S (FL)-reactive B_mem_ in pre-COVID era PBMC samples cannot be attributed to impaired functionality since pan IgG^+^ ASCs (measured irrespective of antigen specificity; refer to Supplementary Figure S1B for an illustration of the assay principle) were readily apparent in these samples. Furthermore, the detection of B_mem_-derived IgG^+^ ASC reactivity against an alternative antigen, a hemagglutinin protein representing the swine-origin H1N1 virus (A/California/04/2009, CA/09) responsible for the 2009 influenza pandemic [63] to which pre-COVID era PBMC donors would have presumable been exposed through natural infection or prophylactic vaccination, serves to further confirm the functionality of these samples.

Thus far, our data indicated that if WH1-S (FL) cross-reactive B_mem_ existed in pre-COVID era samples at all, they occurred at frequencies below the detection limit of our assay. Still, such cross-reactive cells would theoretically have a competitive advantage over naïve B cells if back-boosted by shared epitopes displayed by the WH1-S antigen. Therefore, we next tested whether CCC-reactive B_mem_ increased in frequency in subjects that mounted robust immune responses against the WH1-S protein. Specifically, we utilized a full-length Spike protein representing the NL63 strain of CCC, denoted as NL63-S (FL), to test whether in such PBMC samples an increased frequency of CCC Spike-reactive IgG^+^ ASCs was evident.

**Figure 2:**
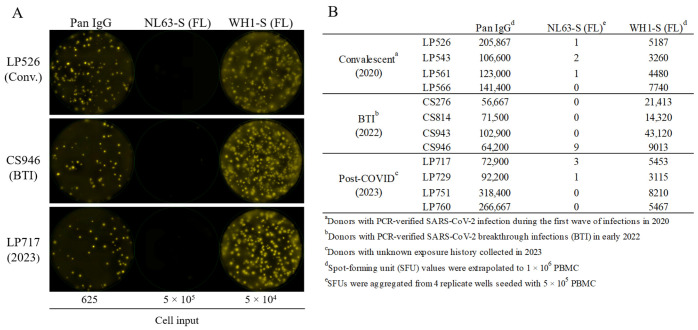
No detectable cross-reactivity against NL63 Spike protein in post-COVID era PBMC samples possessing elevated frequencies of SARS-CoV-2 (WH1) Spike-specific IgG^+^ B_mem_. (**A**) Representative well images depicting FluoroSpot assay results obtained when 5 × 10^5^ polyclonally stimulated PBMCs from post-COVID era donors were input into wells coated with Spike protein representing an *Alphacoronavirus* (denoted as NL63-S). Alternatively, 5 × 10^4^ polyclonally stimulated PBMCs from the same donors were input into wells coated with WH1-S. Pan IgG images are shown (625 PBMCs/well) to confirm the functionality of the PBMC samples following in vitro polyclonal stimulation, respectively. (**B**) Summary table displaying assays results obtained when testing four donors originating from the three defined cohorts.

Well images are shown in Figure 2A, depicting results obtained when testing a representative donor from each of the convalescent, breakthrough infection (BTI), and post-COVID era cohorts. Despite detecting high frequencies of WH1-S (FL)-reactive IgG^+^ ASC in wells plated with 5 × 10^4^ PBMCs, IgG^+^ ASC reactivity against the NL63-S (FL) antigen was completely absent in wells seeded with a ten-fold higher cell input (5 × 10^5^ PBMCs). Results of testing four donors from the different cohorts are summarized in Figure 2B. Notably, despite leveraging the affinity capture coating methodology to ensure high-density antigen coating [58], we did not have access to positive control PBMC samples from donors with known NL63 infections; hence, we could not verify that the NL63-S (FL) protein was conformationally intact or capable of enabling the detection of antigen-specific IgG^+^ ASCs.

Therefore, we expanded our panel of Spike-related antigens to include multiple CCC strains and additionally directed our focus toward the RBD since antibody binding to this region positively correlates with neutralizing activity against the ancestral SARS-CoV-2 strain [64]. We hypothesized that if the WH1-S RBD contains structurally conserved epitopes capable of back-boosting B_mem_ from prior CCC infections, then post-COVID era donors (collected in 2023) would show elevated frequencies of IgG^+^ ASCs with specificity for the RBD of one or more CCCs. Owing to prior observations indicating that affinity capture coating of WH1-S (RBD)-His protein failed to yield well-defined secretory footprints [28], we instead leveraged an alternative detection strategy referred to as the “inverted assay” (the assay principle is illustrated for fluorescence-based and enzymatic-based ImmunoSpot^®^ assays in Supplementary Figures S2 and S3, respectively) for detection of RBD-reactive IgG^+^ ASCs. Notably, despite the detection of elevated numbers of WH1-S (RBD)-reactive IgG^+^ ASCs in this post-COVID era donor cohort, we did not observe appreciable IgG^+^ ASC reactivity against RBD probes representing the 229E, NL63, or HKU1 strains of CCC (Table 1). However, IgG^+^ ASC reactivity against an RBD probe representing the SARS-CoV-2 Omicron (BA.1) variant was detected in these donors—the relevance of this observation will be addressed in greater detail below. Well images depicting results obtained when testing PBMCs from a post-COVID era donor in single-color inverted FluoroSpot assays using His-tagged RBD probes are shown in Supplementary Figure S4. Here again, the absence of positive control PBMC samples with verified exposures to the different CCCs precluded us from definitively concluding that WH1-S (RBD)-reactive B_mem_ were generated independently of cross-reactive back-boosting. Collectively, the data presented here, along with the kinetics of the WH1-S-specific B cell response following the initial inoculation of previously SARS-CoV-2 naïve subjects with COVID-19 mRNA vaccines [17,65,66], suggest that the B cell response engaged by the WH1-S antigen largely originated from a naive repertoire lacking appreciable cross-reactivity for endemic CCCs. However, our subsequent analysis of B cell cross-reactivity amongst SARS-CoV-2 variants present below is independent of this conclusion.

### 3.2. Rationale and Considerations for Performing Dual-Label Inverted ImmunoSpot^®^ Assays

**Figure 3:**
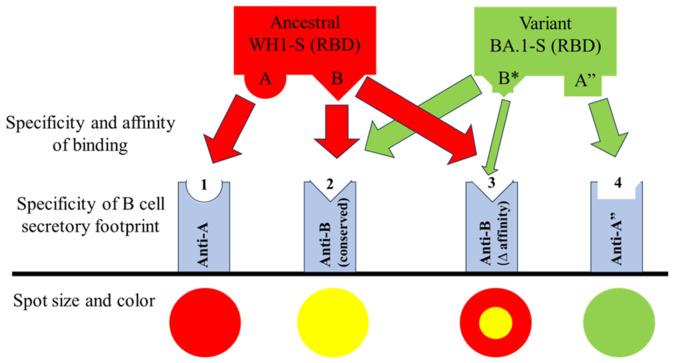
Conceptual framework of how the dual-label ImmunoSpot^®^ assay enables distinction between SARS-CoV-2 strain-specific and cross-reactive secretory footprints. For simplicity, only two epitopes (A and B) are denoted on the ancestral WH1-S (RBD) antigen probe (depicted in red). On the corresponding variant BA.1-S (RBD) antigen probe (depicted in green), epitope A has an alternative conformation (A”) that results in the complete loss of binding to secretory footprints originating from anti-A-specific ASCs. Conversely, the mutated A” epitope represents an antigenic site recognized exclusively by anti-A”-specific ASCs. Owing to lack of cross-reactivity between the A and A” epitopes, when both antigen probes are added (see Supplementary Figure S6 for an illustration of the dual-label inverted B cell ImmunoSpot^®^ test principle), secretory footprints originating from anti-A-specific ASCs will only capture the WH1-S (RBD) antigen probe, whereas only anti-A”-specific ASCs will capture the mutated BA.1-S (RBD) antigen probe. These single-positive (SP) scenarios are denoted as “red only” (1) and “green only” (4) secretory footprints in this schematic. Cross-reactive ASCs that recognize epitope B, which remains conformationally conserved between the ancestral and variant antigens, will capture equivalent amounts of WH1-S (RBD) and BA.1-S (RBD) probes and results in double-positive (DP) spots in which the red and green secretory footprints are completely overlapping (2, depicted as a yellow secretory footprint). However, if an anti-B-specific ASC possesses reduced affinity for the mutated B* epitope on the variant BA.1-S (RBD) relative to the ancestral WH1-S (RBD) probe, the resulting DP secretory footprint will exhibit a larger red secretory footprint due to differences in binding affinity (3).

Having already established single-color inverted assays to enumerate IgG^+^ ASC reactivity using His-tagged RBD probes (WH1 or BA.1), we next sought to test the hypothesis that this assay methodology would also enable distinction between strain-specific and cross-reactive ASCs (refer to Figure 3 and Supplementary Figure S5 for schematic illustrations). Conceptually, an ASC-derived secretory footprint with equivalent affinity for two different RBD probes should capture both simultaneously, resulting in a dual-labeled SFU. Conversely, secretory footprints specific for only one RBD probe would be single-labeled. Furthermore, we anticipated that this dual-label approach would also identify secretory footprints originating from cross-reactive ASC with differential binding affinities for the RBD probes, respectively, based on the overlap of the resulting SFUs.

Secondarily, based on experience from performing single-color inverted assays [28,67], we appreciated the importance of plating polyclonally stimulated samples at donor-specific “Goldilocks” cell inputs (not exceeding 5 × 10^4^ PBMCs per well). This is necessary to (1) prevent local saturation of the capture antibodies’ capacity to ensure optimal secretory footprint formation; and (2) avoid SFU crowding that would preclude single-cell resolution when testing samples with elevated frequencies of antigen-specific ASCs. To this end, we first tested donor PBMCs in single-color inverted assays using His-tagged RBD proteins (refer to Supplementary Figure S6) to confirm the presence of RBD-reactive SFUs, and to define the ideal “Goldilocks” input for subsequent assessments in dual-label inverted FluoroSpot assays.

### 3.3. Establishing the Dual-Label Inverted ImmunoSpot^®^ Assay to Measure ASC Cross-Reactivity at Single-Cell Resolution

To develop an assay capable of incorporating various SARS-CoV-2 variant RBD probes, we first devised a strategy to distinguish between two probes simultaneously. We identified a commercially available WH1-S (RBD) probe with a genetically encoded FLAG tag and sought to determine its suitability for inverted assays, and whether it could detect WH1-S (RBD)-reactive IgG^+^ ASC with the same specificity and sensitivity as the previously used WH1-S (RBD)-His probe. To compare these probes, we performed single-color enzymatic inverted assays using donor PBMCs and equivalent concentrations (100 ng/mL) of either the FLAG- or His-tagged RBD probes (Supplementary Figure S7). Representative images obtained using these probes, along with their corresponding anti-affinity tag detection reagents, are shown in Supplementary Figure S7A. Importantly, these results verified that SFU detection was comparable using either the His- or FLAG-tagged detection probes.

We next transitioned from enzymatic to fluorescence-based assays (FluoroSpot) to measure both probes simultaneously in distinct channels without signal cross-bleed. Because inverted FluoroSpot assays lack enzymatic amplification, the resulting SFU size is directly proportional to the amount of antigen probe retained. Therefore, we re-tested select samples at donor-specific “Goldilocks” cell inputs (yielding ~50 SFUs/well). As shown in Supplementary Figure S7C,D, both the FLAG and His reagent systems yielded equivalent SFU counts in single-color FluoroSpot assays. Collectively, these data verified the utility of the WH1-S (RBD) FLAG detection probe and further established the feasibility of attempting dual-label assays.

**Figure 4:**
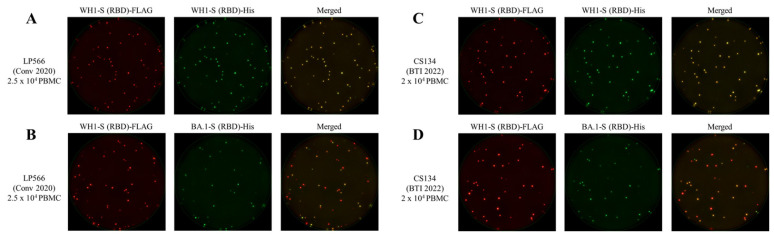
Dual-label inverted FluoroSpot permits the distinction between strain-specific and cross-reactive ASCs. (**A**,**B**) Representative well images depicting dual-label inverted FluoroSpot assay results obtained when polyclonally stimulated PBMCs from a PCR-verified, convalescent (Conv) SARS-CoV-2-infected donor (LP566) were input into wells coated with anti-human IgG Fc-specific capture antibody. In (**panel A**), well images depict the detection of individual spot-forming units (SFUs) using either the WH1-S (RBD)-FLAG or WH1-S (RBD)-His antigen probes. Additionally, the well images were merged (virtual overlay of color planes) using the ImmunoSpot^®^ software to visualize dual-labeling of SFUs. Likewise, in (**panel B**), the same donor is shown but the BA.1-S (RBD)-His RBD antigen representing a future SARS-CoV-2 variant was used in combination with the WH1-S (RBD)-FLAG probe. (**C**,**D**) Representative images depicting results obtained when testing PBMCs from a donor with PCR-verified SARS-CoV-2 infection in early 2022 (breakthrough infection, BTI). In (**panel C**), individual SFUs detected using either the WH1-S (RBD)-FLAG or WH1-S (RBD)-His antigen probes are shown, along with a virtual overlay of the color planes. In (**panel D**), PBMCS from the same donor were tested using the BA.1-S (RBD)-His RBD antigen in combination with the WH1-S (RBD)-FLAG probe. Note: images were contrast enhanced and adjusted for brightness to aid visualization.

Having validated the His- and FLAG-tagged WH1-S (RBD) probes individually, we next verified that their simultaneous use (at 50 ng/mL) permitted the precise dual-labeling of individual SFUs. As shown in Figure 4A, we tested PBMCs from a 2020 convalescent donor (LP566). Nearly all SFUs revealed by the WH1-S (RBD)-FLAG probe (red channel) were dual-labeled with the WH1-S (RBD)-His probe (green channel). In contrast, when the BA.1-S (RBD)-His probe was paired with the WH1-S (RBD)-FLAG probe, fewer SFUs appeared in the His/green channel (Figure 4B). Many of the WH1-S (RBD)-positive SFUs also lacked BA.1 co-labeling, confirming that these probes were antigenically distinct and that the assay could distinguish between WH1-specific ASCs and those recognizing cross-reactive epitopes on BA.1-S (RBD). Figure 4C,D depicts similar dual-label assays using PBMCs from a COVID-19 mRNA-vaccinated donor (CS134) who had a breakthrough infection (BTI) in January 2022. As expected, nearly all WH1-S (RBD)-FLAG SFUs were dual-labeled when paired with the WH1-S (RBD)-His probe. However, when using the BA.1-S (RBD)-His probe, we observed an increased frequency of co-labeled SFUs compared to the 2020 donor. This indicates that a higher proportion of ASCs from this BTI donor recognized cross-reactive epitopes on the BA.1-S (RBD) probe. The implications of this observation will be elaborated upon in greater detail below.

Lastly, to stringently evaluate the assay’s ability to discern strain-specific and cross-reactive ASCs, we incorporated two additional His-tagged RBD probes representing Omicron lineage variants (JN.1 and XFG) with progressively greater antigenic distances from the ancestral WH1 strain [68,69] (Supplementary Figure S8). Both probes successfully detected SFUs in PBMC samples collected in 2025. Notably, in both donors, the WH1-S (RBD)-FLAG probe detected a higher number of SFUs than either the BA.1, JN.1, or XFG His-tagged probes. In aggregate, these data support the feasibility of using the dual-label inverted assay to characterize the specificity and cross-reactivity of SARS-CoV-2 Spike (RBD)-reactive B_mem_ repertoires across diverse donor cohorts.

### 3.4. Assessing the Specificity and Cross-Reactivity of SARS-CoV-2 Spike (RBD)-Reactive B_mem_ in Well-Defined Donor Cohorts

A fundamental question in vaccinology, and specifically in the context of mutable pathogens such as SARS-CoV-2, is whether an individual’s first exposure results in “immunological imprinting”, and whether subsequent responses to the same or related antigens will focus primarily on conserved antigenic determinants [45,70]. It is now evident that the SARS-CoV-2 virus has undergone continuous antibody-mediated antigenic drift since entering the human population [71,72]. Furthermore, immune-mediated selection pressure has led to the accumulation of amino acid alterations in the RBD region, enabling escape from neutralizing antibody activity acquired either through passive transfer of monoclonal antibodies, prior infection, and/or COVID-19 vaccination [44,73,74,75,76,77,78]. Consequently, in recent years, COVID-19 vaccines have been modified on several occasions with the intent of eliciting immune responses conferring superior breadth of protection against emerging variants relative to those achieved following booster immunization with the ancestral WH1 strain. However, it remains unclear if additional exposures to variant Spike proteins—either through infection and/or vaccination—will elicit de novo B_mem_ responses that are variant-specific, or whether recall responses will remain predominantly focused on antigenic determinants shared with the ancestral WH1 strain.

To this end, we utilized dual-label inverted assays to systematically characterize the antigen-reactivity profile of B_mem_-derived IgG^+^ ASCs following polyclonal stimulation of PBMCs originating from three defined cohorts (refer to Section 2.1 and Supplemental Table S1). To characterize hundreds of individual secretory footprints at single-cell resolution, we plated PBMCs at donor-specific “Goldilocks” inputs (not exceeding 5 × 10^4^ cells/well) into replicate wells. These were subsequently developed with various combinations of WH1-S (RBD)-FLAG and variant RBD-His detection probes. Using machine-assisted automated counting, we then enumerated the SFUs detected in the red (FLAG) and green (His) channels and measured their footprint sizes to facilitate high-content analysis. Merging the data from replicate wells into a single flow cytometry standard (FCS) file permitted the use of FlowJo^™^ to segregate SFU “events” into three broad categories: WH1-S (RBD)-FLAG single-positive (SP), Variant RBD-His SP, and WH1-S + RBD-His double-positive (DP). Within the DP population, we sought to further distinguish SFUs based on whether their red or green footprints were larger in either channel, or whether they were proportionally equivalent since this provides insights into their binding affinities for the ancestral WH1 versus variant RBD probes.

**Figure 5:**
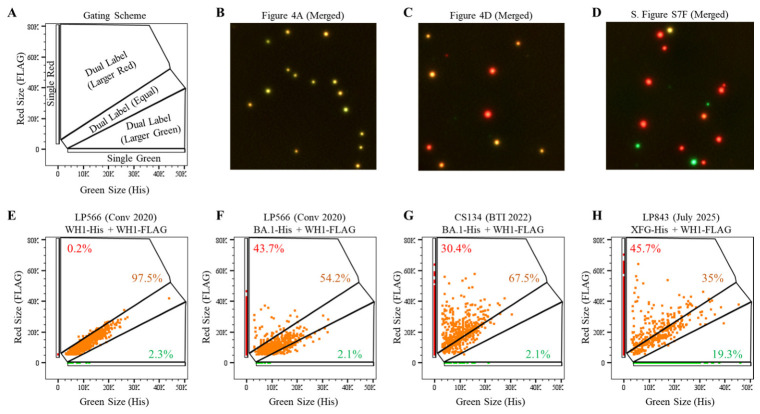
High-content data analysis of dual-label inverted FluoroSpot assay results. (**A**) Schematic representation of the flow cytometry standard (FCS) plot and corresponding gates used for segregating single- (Red or Green) and dual-labeled (Red + Green) spot-forming units (SFUs) detected in inverted FluoroSpot assays based on the size of the corresponding secretory footprints. Gates further segregating dual-labeled (double-positive, DP) secretory footprints into those that were larger red or larger green; those that were proportionally equivalent in size are also denoted. (**B**–**D**) Magnification of representative well images in which the red and green fluorescence detection planes were merged (virtual overlay) using the ImmunoSpot^®^ software. (**Panel B**) is an enlarged region of a merged well image (refer to Figure 4A) depicting uniform dual-labeling of SFUs using antigenically matched WH1-S (RBD)-FLAG and WH1-S (RBD)-His probes. (**Panel C**) is an enlarged region of the merged well image shown in Figure 4D depicting dual-labeled SFU in which the red secretory footprints revealed by the WH1-S (RBD)-FLAG probe were larger than the secretory footprint revealed using the BA.1-S (RBD)-His probe. (**Panel D**) is an enlarged region of the merged well image shown in Supplementary Figure S8F depicting single- and dual-labeled secretory footprints, which were revealed using the combination of WH1-S (RBD)-FLAG and XFG-S (RBD)-His probes. (**E**,**F**) FCS plots depicting the segregation of SFUs generated by LP566 (collected in 2020) using either WH1-S (RBD)-His (**panel E**) or BA.1-S (RBD)-His (**panel F**) antigen probes in combination with WH1-S (RBD)-FLAG antigen probe. (**G**) FCS plot depicting the segregation of SFUs generated by CS134 (collected in 2022) using the combination of WH1-S (RBD)-FLAG and BA.1-S (RBD)-His probes. (**H**) FCS plot depicting the segregation of SFUs generated by LP843 (collected in July 2025) using the combination of WH1-S (RBD)-FLAG and XFG-S (RBD)-His probes. Note: the relative frequency of single-red, dual-labeled or single-green SFUs amongst all counted secretory footprints for each donor and RBD antigen probe combinations are denoted in panels (**E**–**H**), respectively.

The gating strategy used for our dual-label analysis is shown in Figure 5A (see also Supplementary Figure S9). Upon magnification of well images in which the red and green channels were virtually overlaid (merged), each of the individual SFUs was proportionally co-labeled with the WH1-S (RBD)-FLAG and WH1-S (RBD)-His probes (Figure 5B) and served as an internal control for proportionally equivalent SFUs. In contrast, Figure 5C depicts an alternative scenario in which individual SFUs possessed larger secretory footprints in the FLAG (red) compared to the His (green) detection channel; these footprints appear red with yellow centers, denoting the region of signal co-localization. Lastly, Figure 5D provides a representative image of SFUs detected solely in the His (green) channel. Collectively, these enlarged well images verify that distinct SFU categories are detectable in dual-label inverted FluoroSpot assays and substantiate the gating scheme defined in Figure 5A.

Consistent with the visual observation of proportional co-labeling, Figure 5E depicts an FCS plot from the representative 2020 convalescent donor (LP566), confirming that nearly all SFUs detected with the WH1-S (RBD)-FLAG and WH1-S (RBD)-His probes resided in the DP gate. Moreover, these DP SFUs were predominantly restricted to the region with equivalent footprint sizes in both channels. Figure 5F shows the results for the same 2020 donor when evaluated with the WH1-S (RBD)-FLAG and BA.1-S (RBD)-His probes. While approximately half of the SFUs were cross-reactive (DP), the remaining half were classified as WH1-S (RBD) strain-specific (SP). Notably, there was no evidence of BA.1-S (RBD)-specific ASCs in this donor. Similarly, the representative BTI donor (CS134) also lacked BA.1-S (RBD)-specific ASCs (Figure 5G). Instead, this donor showed an increased frequency of DP SFUs, many of which exhibited larger footprints in the FLAG (WH1) channel. FCS plots depicting this donor’s PBMCs (along with LP843, see below) using additional combinations of RBD probes are shown in Supplemental Figure S11. Lastly, Figure 5H displays data from a post-COVID era donor (LP843) tested with WH1-S (RBD)-FLAG and XFG-S (RBD)-His probes. While the largest fraction of SFUs were WH1-S (RBD) SP, a sizeable frequency of XFG-S (RBD) SP SFUs was also detected, supporting the de novo generation of variant-specific B_mem_.

**Figure 6:**
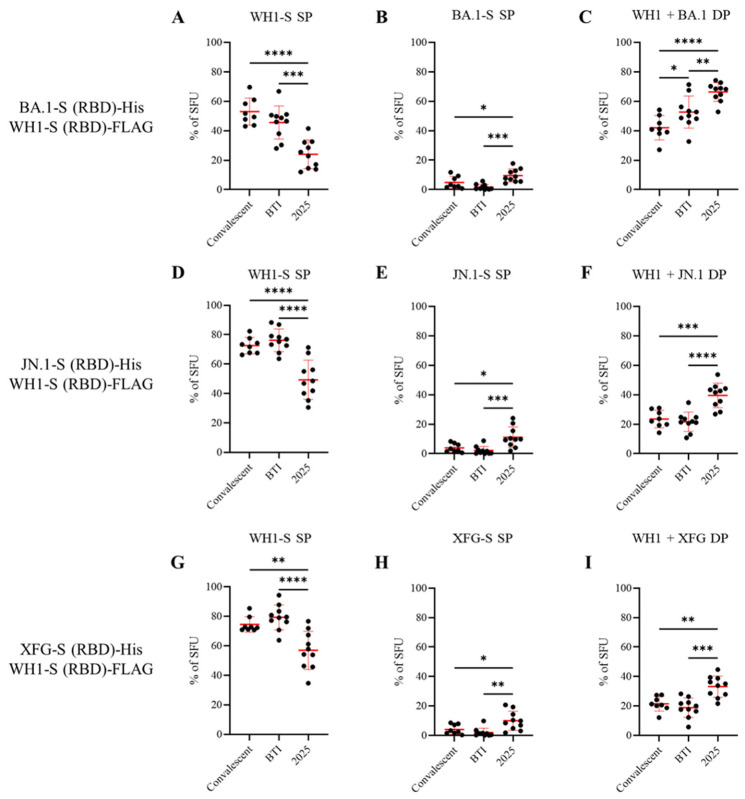
Frequency of single-positive (SP) and double-positive (DP) SARS-CoV-2-specific SFUs revealed using WH1-S (RBD)-FLAG and variant (RBD)-His antigen probes. Polyclonally stimulated PBMCs originating from donors that (*i*) recovered from PCR-verified SARS-CoV-2 infections in 2020 (convalescent) prior to the availability of COVID-19 vaccines (*n* = 8), (*ii*) recovered from PCR-verified SARS-CoV-2 infection in early 2022 (breakthrough infection, BTI) (*n* = 10), or (*iii*) collected in 2025 without defined histories of past infection(s) or vaccination(s) (*n* = 10) were evaluated in dual-label inverted ImmunoSpot^®^ assays (refer to Section 2.4.5). (**A**–**C**) Frequency of SP or DP secretory footprints detected using the combination of WH1-S (RBD)-FLAG and BA.1-S (RBD)-His antigen probes. (**D**–**F**) Frequency of SP or DP secretory footprints detected using the combination of WH1-S (RBD)-FLAG and JN.1-S (RBD)-His antigen probes. (**G**–**I**) Frequency of SP or DP secretory footprints detected using the combination of WH1-S (RBD)-FLAG and XFG-S (RBD)-His antigen probes. Statistical significance (* *p* < 0.05, ** *p* < 0.01, *** *p* < 0.001, **** *p* < 0.0001) of differences between donor cohorts were determined using Welch’s analysis of variance (ANOVA) test with Dunnett’s T3 post hoc correction for multiple comparisons.

Extending these analyses to the three defined cohorts, namely convalescent, BTI and post-COVID era donors collected in 2025, Figure 6A–C portrays the segregation of SP and DP SFUs detected using the combination of WH1-S (RBD)-FLAG and BA.1-S (RBD)-His probes. Compared to the 2025 cohort, both the convalescent (mean difference 29, 95% CI [17 to 41]) and BTI (mean difference 21.5, 95% CI [9.2 to 34]) cohorts exhibited significantly (*p* < 0.001) increased frequencies of WH1-S (RBD) SP SFUs (Figure 6A). Additionally, the 2025 cohort possessed a significantly (mean difference 7.9, 95% CI [3.7 to 12], *p* < 0.001) increased frequency of BA.1-S (RBD) SP SFUs compared to the BTI cohort collected in early 2022 (Figure 6B) despite such donors having PCR-verified infections, presumably caused by an Omicron variant strain, prior to sample collection. Moreover, the 2025 cohort also demonstrated a significantly increased frequency of WH1 + BA.1 DP SFUs compared to the convalescent (mean difference 24, 95% CI [15 to 34], *p* < 0.001) and BTI donor cohorts (mean difference 14, 95% CI [2.9 to 24], *p* < 0.05) (Figure 6C). Using the combination of WH1 and JN.1 RBD probes, we detected a significant (*p* < 0.001) reduction in the frequency of WH1 SP SFUs in the 2025 cohort compared to the convalescent (mean difference 23, 95% CI [11 to 36]) and BTI (mean difference 27, 95% CI [14 to 40]) cohorts (Figure 6D). The 2025 cohort also possessed a significantly increased frequency of JN.1 SP SFUs compared to the BTI (mean difference 9, 95% CI [2.5 to 16], *p* < 0.01) and convalescent (mean difference 7.3, 95% CI [0.6 to 14], *p* < 0.05) donor cohorts (Figure 6E). Among the three cohorts, 2025 donors possessed a significantly (*p* < 0.001) higher frequency of WH1 + JN.1 DP SFUs compared to the convalescent (mean difference 16, 95% CI [7.1 to 25]) and BTI (mean difference 18, 95% CI [9 to 27]) donor cohorts (Figure 6F). Using the combination of WH1 and XFG RBD probes, the 2025 donors continued to demonstrate a reduced frequency of WH1 SP SFUs compared to the convalescent (mean difference 18, 95% CI [5.2 to 30], *p* < 0.01) and BTI (mean difference 22, 95% CI [9.4 to 35], *p* < 0.001) donor cohorts (Figure 6G). 2025 donors also possessed a significantly (mean difference 8, 95% CI [1.9 to 14], *p* < 0.01) increased frequency of XFG SP SFUs compared to the BTI cohort (Figure 6H). Lastly, the 2025 cohort exhibited a higher frequency of WH1 + XFG DP SFUs compared to the convalescent (mean difference 12, 95% CI [4 to 19], *p* < 0.01) or BTI (mean difference 14, 95% CI [6.2 to 22], *p* < 0.001) donor cohorts (Figure 6I). Similar analysis was also performed for the combination of WH1-S (RBD)-FLAG and WH1-S (RBD)-His probes, and the vast majority of SFUs were DP for all cohorts (Supplementary Figure S10). Highlighting the depth of coverage achieved for each donor in the dual-label inverted assays, Supplementary Table S4 denotes the cumulative SFU counts detected in the SP and DP categories for each of the RBD probe combinations tested. Moreover, since an equivalent number of replicate wells were merged for each donor and RBD probe combination, the cumulative SFU counts also demonstrate the shift in their distribution. Collectively, these data illustrate how the dual-label inverted FluoroSpot methodology—combined with automated counting and downstream analysis—facilitated the interrogation of IgG^+^ ASC reactivity at single-cell resolution against antigenically distinct SARS-CoV-2 RBDs and the detection of elevated frequencies of cross-reactive and Omicron lineage-specific B_mem_ responses in PBMC samples collected in 2025 compared to the convalescent and BTI cohorts.

### 3.5. High-Content Analysis of DP SFUs Provides Evidence for Back-Boosting of Cross-Reactive B_mem_ Following BTI

**Figure 7:**
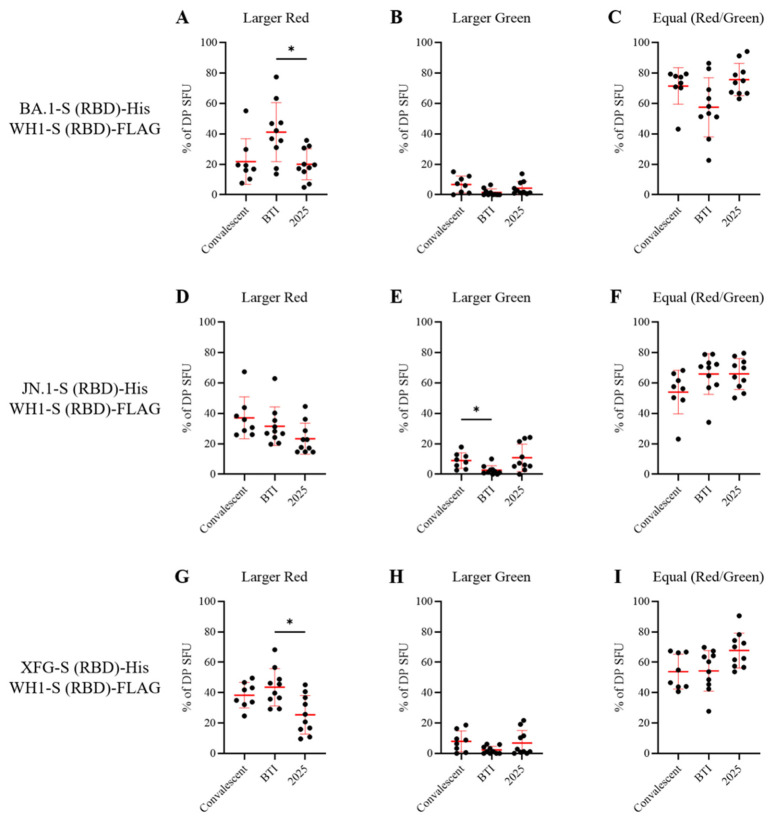
Frequency of double-positive SFUs with distinct secretory footprints sizes in dual-label inverted ImmunoSpot^®^ assays. Double-positive (DP) secretory footprints detected in the dual-label inverted ImmunoSpot assay were further segregated into three categories based on the size of the secretory footprints revealed using the WH1-S (RBD)-FLAG (red detection channel) in combination with His-tagged RBD antigens (green detection channel) representing SARS-CoV-2 variants (refer to Figure 5A for gating strategy). (**A**–**C**) Frequency of DP secretory footprints co-labeled with WH1-S (RBD)-FLAG and BA.1-S (RBD)-His antigen probes that were larger red (**panel A**), larger green (**panel B**), or proportionally equivalent in size (**panel C**). (**D**–**F**) Frequency of DP secretory footprints co-labeled with WH1-S (RBD)-FLAG and JN.1-S (RBD)-His antigen probes that were larger red (**panel D**), larger green (**panel E**), or proportionally equivalent in size (**panel F**). (**G**–**I**) Frequency of DP secretory footprints co-labeled with the WH1-S (RBD)-FLAG and XFG-S (RBD)-His antigen probes that were larger red (**panel D**), larger green (**panel E**), or equal in size (**panel F**). Statistical significance (* *p* < 0.05) of differences between donor cohorts were determined using Welch’s analysis of variance (ANOVA) test with Dunnett’s T3 post hoc correction for multiple comparisons.

Beyond enumerating the frequency of DP SFUs (Figure 5 and Supplementary Figure S11), we further sought to segregate the cross-reactive (DP) population into those exhibiting larger footprints for a particular RBD probe and those with proportionally equivalent secretory footprint sizes in both detection channels. Namely, because detection of a larger secretory footprint in one channel signifies increased capture/retention of that probe and hence preferential binding affinity, we were particularly interested in determining if donors with PCR-verified BTI exhibited evidence for WH1-dominant DP SFUs owing to prior immunological imprinting and back-boosting of B_mem_ that possessed a high affinity for the ancestral WH1 strain relative to the (presumed to be BA.1) variant responsible for infection. As shown in Figure 7A, this was indeed the case: the BTI cohort possessed a significantly (mean difference 21, 95% CI [2.5 to 40], *p* < 0.05) higher frequency of DP SFUs that were larger in the WH1 (red/FLAG) channel compared to the convalescent or 2025 cohorts. Notably, neither the BTI nor 2025 cohorts exhibited an increased frequency of DP SFUs that were larger in the BA.1 (green/His) channel compared to the convalescent donors (Figure 7B). However, we did detect a high frequency (mean difference 18, 95% CI [0.6 to 37], *p* = 0.0592) of DP SFUs with proportionally equivalent secretory footprint sizes in the 2025 cohort relative to the BTI donors (Figure 7C). Figure 7D–F depicts similar analysis of DP SFUs detected using the combination of WH1 and JN.1 RBD probes. Figure 7G–I depicts the same analysis using the combination of WH1 and XFG RBD probes. Compared to the 2025 cohort, the BTI donors exhibited a significantly (mean difference 18, 95% CI [3.5 to 33], *p* < 0.05) higher frequency of DP SFUs that were larger in the WH1 (red/FLAG) channel (Figure 7G). Supplementary Table S5 specifies the SFU counts detected in the three DP categories, respectively, for each of the RBD probe combinations tested. Again, since an equivalent number of replicate wells were merged for each donor and RBD probe combination, the cumulative SFU counts in the three subcategories also demonstrate the shift in their distribution. In aggregate, our high-content analysis of DP SFUs further substantiates, at single-cell resolution, our observations of “original antigenic sin” in the BTI cohort. Furthermore, these data also demonstrate that the B_mem_ repertoire in the 2025 cohort possessed both increased breadth and enhanced affinity for contemporary SARS-CoV-2 variants.

## 4. Discussion

Using the ImmunoSpot^®^ methodology, we evaluated PBMCs from defined cohorts for B_mem_ reactivity against Spike proteins representing CCCs and SARS-CoV-2. In pre-COVID era samples, frequencies of SARS-CoV-2 Spike “reactive” B_mem_ were below the assay’s limit of detection (Supplementary Table S2). Our data demonstrate a lack of appreciable back-boosting among IgG^+^ B_mem_ that recognize structurally conserved epitopes shared between CCCs and SARS-CoV-2. Specifically, despite verifying elevated frequencies of SARS-CoV-2 Spike-specific IgG^+^ ASC activity in post-COVID era donors, we did not detect IgG^+^ activity against the NL63 Spike (Figure 2). Conversely, a recent publication found evidence that B_mem_ recognizing shared epitopes in the S2 domain were back-boosted in COVID-19 patients following severe infection [79]. We also developed a novel dual-label inverted FluoroSpot methodology that distinguished between strain-specific and cross-reactive IgG^+^ ASCs. The flexibility of this system allowed us to substitute different His-tagged antigen probes to systematically assess IgG^+^ ASC reactivity against Omicron variants that were progressively more antigenically distinct from the ancestral WH1 strain. Furthermore, our high-content analysis of secretory footprint sizes in the respective detection channels provided insights into the relative binding affinity of individual ASCs for the ancestral and variant RBD probes.

Antigenic imprinting, historically referred to as “original antigenic sin,” was first described by Thomas Francis Jr. during studies of antibody responses to influenza virus vaccination [80]. Both terms describe the propensity of the immune system to preferentially recall pre-existing B_mem_ that recognize structurally conserved determinants shared between the “original” priming antigen and a “drift variant.” This recall often occurs to the detriment of mounting de novo, strain-specific immune responses. Driven by continuous immune pressure since its emergence, the SARS-CoV-2 Omicron lineage has acquired numerous amino acid substitutions and alterations in the Spike protein—particularly within the receptor-binding domain (RBD)—that facilitate escape from neutralizing antibody activity. Consequently, COVID-19 vaccines have been updated repeatedly to elicit immune responses that confer a broader breadth of protection against newly emerging SARS-CoV-2 variants.

To this end, data generated by our dual-label inverted assays align with numerous studies demonstrating preferential back-boosting of B_mem_ that recognize shared determinants between the ancestral WH1 Spike protein and Omicron variants [42,43,81,82,83]. Notably, when evaluating PBMC samples collected in 2025, our dual-label assays revealed strong evidence for the generation of Omicron strain-specific B_mem_ in several donors. These findings are consistent with data from Yisimayi and colleagues [84], suggesting that multiple exposures to the antigenically distinct Omicron Spike are required to drive the expansion of strain-specific B_mem_. Furthermore, our high-content analysis of dual-labeled spot-forming units (SFUs) from individuals with 2022 breakthrough infections—each representing the secretory footprint of a single ASC—corroborates the detailed analysis performed by Wang and colleagues [85]. Both datasets demonstrate that these B_mem_ retain a higher affinity for the ancestral WH1 strain than for Omicron subvariants. In aggregate, our data demonstrates how the dual-label inverted assay methodology yields independent, single-cell level validation for findings traditionally generated through more laborious alternative techniques, such as antigen probe-based flow cytometry, serum antibody depletion, and characterization of individual mAbs. Relatedly, we did not evaluate culture supernatant samples from the polyclonal stimulation cultures for the presence of SARS-CoV-2 RBD-reactive IgG, or neutralizing activity for the ancestral WH1 or Omicron variant strains, owing to the low abundance of antigen-specific IgG and because such data would not permit distinction between antibody activity that was strain-specific vs. broadly reactive.

Notwithstanding our key observations, this study has several limitations. First, we lacked positive control PBMC samples from donors with verified, recent seasonal common cold coronavirus (CCC) infections. Additionally, because we utilized a single full-length trimeric Spike protein representing the NL63 strain to detect cross-reactive ASCs, we cannot definitively confirm the functionality of this specific protein within our assay system. Notably, cross-reactive B_mem_ clones targeting structurally conserved epitopes in the S2 region of CCCs reportedly outnumber seasonal, CCC strain-specific clones in the post-COVID era [86]. Yet, we detected negligible ASC reactivity in our own assays despite testing two million PBMCs (Figure 2).

Second, the sample size of the convalescent (*n* = 8) and breakthrough infection (BTI) (*n* = 10) cohorts were limited. Moreover, our dual-label studies did not include a cohort of COVID-19-vaccinated subjects without prior SARS-CoV-2 infection. While such samples were tested in our prior publication [28], cryopreserved PBMCs were no longer available for these donors to permit their inclusion in the present study. Consequently, our findings may not fully reflect the heterogeneity in magnitude or quality of immune responses elicited following these SARS-CoV-2 infection scenarios. Relatedly, our BTI samples were collected exclusively during the acute phase following PCR-verified infections—ranging from 13 to 36 days post-diagnosis (Supplementary Table S1)—with no longitudinal follow-up. Consequently, it remains unclear whether these donors eventually developed higher frequencies of Omicron-specific B_mem_ over time. Furthermore, the precise history of SARS-CoV-2 antigen exposure for our 2025 cohort was unknown. Therefore, we were unable to attribute the induction of Omicron-specific (SP) SFUs to specific vaccination or infection events. Despite this, we observed a trend toward increased Omicron-specific (SP) SFUs in dual-label inverted assays when testing PBMC samples collected later in 2025 (Supplementary Table S4). Finally, due to the lack of suitable FLAG-tagged RBD probe representing the BA.1 or JN.1 strains, our assays could not resolve ASCs that were singly specific for individual Omicron subvariants. However, given the inherent flexibility of utilizing the His and FLAG affinity tags for RBD probe detection, such dual-label inverted assays can be easily executed once the appropriate reagents become available.

As demonstrated here, the His/FLAG detection system provides exceptional flexibility for the design of dual-label inverted assays. In this study, we selected strategic RBD probes representing antigenically distinct SARS-CoV-2 isolates to systematically assess our donor cohorts. However, future applications of this methodology could seamlessly integrate alternative RBD probes, including updated vaccine strains or newly emerging variants of concern. Likewise, these dual-label inverted assays could utilize variant RBD probes possessing specific amino acid substitutions for epitope mapping, as well as for quantifying the exact proportions of epitope-specific ASCs. Because each SFU originates from a single ASC under optimal assay conditions, this platform offers a powerful, high-throughput alternative to the laborious process of generating and characterizing mAbs from individual donors.

More generally, we contend that the inverted FluoroSpot assay methodology described in this communication has broader applications beyond studying B_mem_ reactivity against SARS-CoV-2 RBD probes. Specifically, ongoing studies in our group are utilizing this platform to dissect B_mem_ reactivity against the hemagglutinin (HA) glycoprotein of influenza A (H1 or H3) viruses. By deploying multimerized detection probes representing distinct HA antigens—which do not require amino acid mutations to ablate sialic acid binding [87]—in tandem with an instrument possessing expanded capabilities, we have successfully scaled the complexity of this methodology. This assay method allows us to dissect the HA-specific ASC repertoire into several distinct subsets exhibiting unique antigen-reactivity profiles. Notably, we incorporated the computationally optimized broadly reactive antigen (COBRA) “P1” HA [88] into our panel of H1 probes, revealing that pre-existing B_mem_ cross-reactivity for this antigen is already present in the human population (Kirchenbaum, unpublished data).

Whereas traditional multiplexed B cell FluoroSpot assays distinguish between ASCs producing different Ig classes or IgG subclasses, we demonstrate how the inverted assay approach enables simultaneous interrogation of ASC reactivity against multiple antigen probes. Beyond segregating strain-specific and cross-reactive ASCs, the inverted assay technique could also be applied for screening donor PBMCs for spontaneous reactivity against allergen panels, potentially offering a more sensitive and reliable alternative to current diagnostics. Likewise, tracking of distinct populations of antigen-specific ASCs following multi-component vaccination (e.g., seasonal influenza vaccines) would provide invaluable insights into their relative immunogenicity while minimizing cell sample requirements. Additionally, utilizing Ig class- or IgG subclass-specific capture antibodies enables selective study of rare B_mem_ or in vivo-differentiated plasmablasts and plasma cells. This is particularly valuable for evaluating ASC populations that are otherwise obscured by dominant populations, such as SARS-CoV-2 Spike-specific IgA^+^ or IgG4^+^ ASCs. Lastly, simple titration of the antigen probe concentration allows investigators to interrogate the relative functional affinity of an evolving ASC repertoire, such as following booster vaccination [67].

## 5. Conclusions

Collectively, the inverted dual-label FluoroSpot methodology presented here provides a powerful alternative to traditional techniques for detecting antigen-specific ASCs and characterizing their cross-reactivity profiles. Owing to the simplicity of antigen probe incorporation, along with recent optimizations in assay conditions, detection reagents, and downstream software-assisted image analysis tools, the inverted ImmunoSpot^®^ assay platform is ideally suited for testing large numbers of samples in a high-throughput workflow. Importantly, such capabilities are essential for dissecting complex B_mem_ and plasmablast responses that arise because of immunological imprinting. This assay platform is therefore uniquely positioned to evaluate B cell responses against rapidly evolving, mutable viruses like SARS-CoV-2 and seasonal influenza, particularly when antigenic drift necessitates vaccine reformulations.

## Figures and Tables

**Table 1:** Lack of shared epitopes between the receptor binding domain (RBD) encoded by cold-causing coronaviruses (CCCs) and SARS-CoV-2. PBMCs from post-COVID era donors collected in 2023 (see Supplementary Table S1) were polyclonally stimulated for five days to convert resting B_mem_ into ASCs. Subsequently, donor PBMCs were evaluated in single-color fluorescent, antigen-specific ImmunoSpot^®^ assays (see Supplementary Figure S2A for an illustration of the assay principle) using His-tagged probes representing the RBD expressed by CCCs or SARS-CoV-2. Data are expressed as spot-forming units (SFUs) per 10^5^ PBMCs. Notably, despite detecting elevated frequencies of antigen-specific IgG^+^ ASCs using RBD probes representing the ancestral (WH1) and Omicron (BA.1) SARS-CoV-2 strains, very few IgG^+^ ASCs were measured using equivalent assay conditions and RBD probes representing three distinct CCC strains.

	6xHis ^a^	229E-S (RBD) ^a,b^	NL63-S (RBD) ^a,b^	HKU1-S (RBD) ^a,c^	WH1-S (RBD) ^a,c,d^	BA.1-S (RBD) ^a,c,e^
LP722	0	1	4	2	223	135
LP724	0	2	1	1	81	55
LP726	0	1	1	3	67	24
LP729	0	0	0	1	354	236
LP731	0	0	2	0	20	26
LP735	0	0	0	0	18	24
LP739	0	2	5	1	31	18
LP751	1	1	2	4	180	99
LP758	0	1	4	1	80	35
LP760	3	0	0	5	93	102
LP761	0	1	9	4	38	24
LP769	2	3	2	4	34	30

^a^ Spot-forming units (SFU) values per 10^5^ PBMCs, ^b^ Member of the genus Alphacoronavirus, ^c^ Member of the genus Betacoronavirus, ^d^ Ancestral SARS-CoV-2 strain (Wuhan-Hu-1, WH1), ^e^ Omicron variant of SARS-CoV-2, Pango lineage B.1.1.529.

## Data Availability

The data generated in this study will be made available by the authors, without undue reservation, to any qualified researcher.

## Supplementary Materials

The following supporting information can be downloaded at: https://www.mdpi.com/article/10.3390/vaccines14070604/s1, Figure S1: Illustration of antigen-specific and pan (total) B cell ImmunoSpot^®^ test principles; Figure S2: Illustration of single-color fluorescent, antigen-specific inverted B cell ImmunoSpot^®^ test principle; Figure S3: Illustration of single-color enzymatic, antigen-specific inverted B cell ImmunoSpot^®^ test principle; Figure S4: No evidence for B_mem_-derived IgG^+^ ASC cross-reactivity between the receptor binding domain (RBD) of cold-causing coronaviruses (CCCs) and SARS-CoV-2; Figure S5: Illustration of dual-label fluorescent, antigen-specific inverted B cell ImmunoSpot^®^ test principle; Figure S6: Testing strategy for determining donor-specific “Goldilocks” cell input for dual-label inverted ImmunoSpot^®^ assays; Figure S7: Single-color enzymatic or fluorescent inverted ImmunoSpot^®^ assays provide comparable sensitivities for detection of WH1-S (RBD)-specific IgG^+^ B_mem_ irrespective of the affinity tag; Figure S8: Dual-label inverted FluoroSpot permits distinction between strain-specific and cross-reactive ASCs; Figure S9: Extended gating scheme for analysis of inverted FluoroSpot data; Figure S10: Frequency of single-positive (SP) and double-positive (DP) SARS-CoV-2-specific SFU revealed using WH1-S (RBD)-FLAG and WH1-S (RBD) His antigen probes; Figure S11: High-content data analysis of dual-label inverted FluoroSpot assay results; Table S1: Donor demographics; Table S2: Lack of detectable B_mem_-derived IgG^+^ ASC reactivity against the ancestral SARS-CoV-2 Spike (WH1-S) protein in pre-COVID era donors; Table S3: Post-COVID era donors possess variable frequencies of SARS-CoV-2 (WH1)-S-specific IgG^+^ B_mem_; Table S4: Cumulative number of single-positive (SP) and double-positive (DP) SFUs detected in dual-label inverted ImmunoSpot^®^ assays; Table S5: Cumulative number of double-positive (DP) SFUs with distinct secretory footprints sizes in dual-label inverted ImmunoSpot^®^ assays.
