# Supplementary material for: Evaluating Memory B Cell Cross-Reactivity Between Ancestral and Future SARS-CoV-2 Variants—Evidence for Original Antigenic Sin
Source: Vaccines (Basel). 2026 Jul 9;14(7):604. doi: 10.3390/vaccines14070604 (PMC13417495; doi:10.3390/vaccines14070604)
Supplement: Supplementary file 1 [file vaccines-14-00604-s001.zip › vaccines-4389764_revised_Supplementary Materials.pdf]

Supplementary Materials

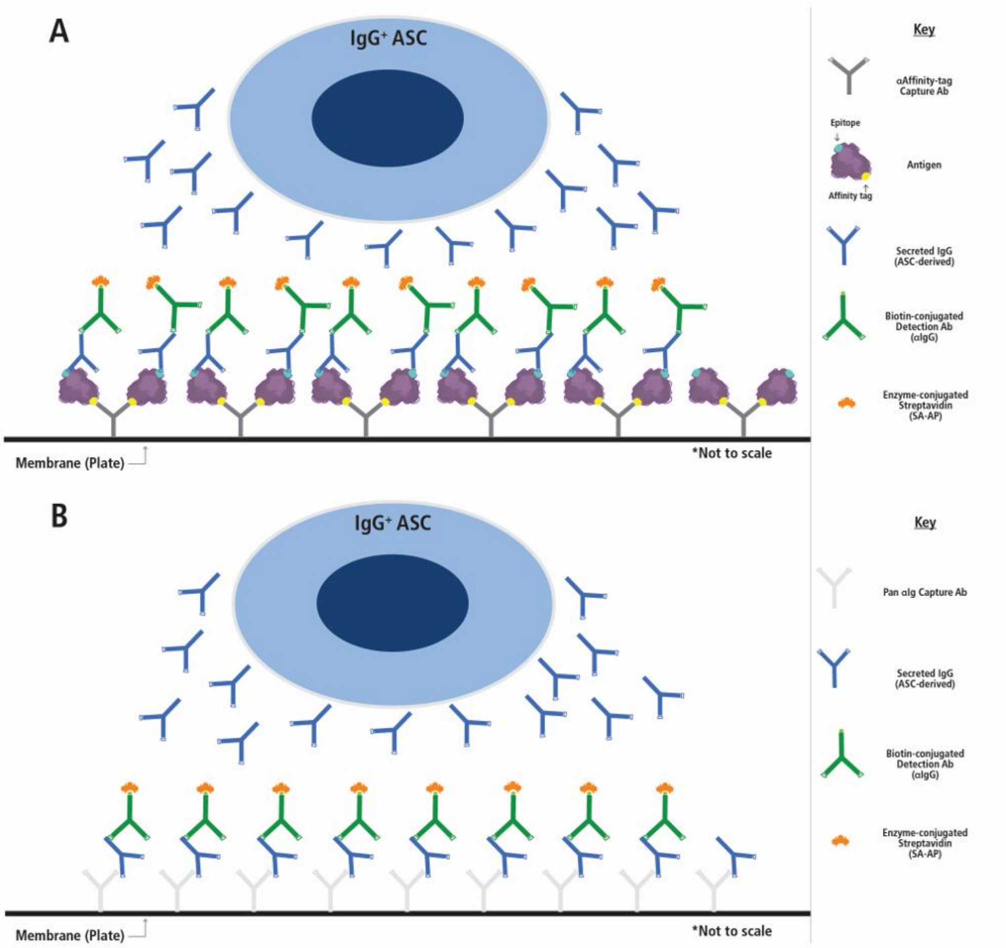

Figure S1: Illustration of antigen-specific and pan (total) B cell ImmunoSpot® test principles. A) In an antigen-specific ImmunoSpot® assay the membrane can be coated directly (not shown) or via affinity capture (as shown) with the antigen of interest to achieve high density antigen coating and maximal detection of antigen-specific antibody-secreting B cells (ASCs)[58]. When ASCs are plated onto such an antigen-coated surface, the antibodies produced by an antigen-specific ASC (shown in blue), as opposed to antibodies produced by ASCs specific to other antigens, will be retained on the membrane in close proximity to where the ASC resided on the membrane as a secretory footprint. Alternatively, in a pan (total) IgG detection assay the Ig produced by antibody-secreting B cells (ASCs) is captured irrespective of its antigen specificity by an anti-species antibody coated onto the membrane (e.g. goat anti-mouse Igκ, depicted in gray) in close proximity to where the ASC resided on the membrane instead of being secreted into the culture supernatant. In both (A) antigen-specific or (B) pan IgG ImmunoSpot® assays, plate-bound IgG-derived secretory footprints are visualized using an anti-human IgG-specific (depicted in red), respectively, followed by deposition of a precipitating visible substrate in an ELISPOT (as shown). Alternatively, the detection antibody could be fluorophore-conjugated and the secretory footprints visualized through selective excitation and measurement of the resulting fluorescence using a suitable instrument (FluoroSpot, not shown).

Table S2: Lack of detectable B_{mem}-derived IgG^+ ASC reactivity against the ancestral SARS-CoV-2 Spike (WH1-S) protein in pre-COVID era donors

		WH1-S (FL)[a,b]	Pan IgG[c]	CA/09 rHA[d,e]
Pre-COVID	LP315	0	132,800	>10/6.25 × 10^4
	LP326	0	209,600	>10/1.25 × 10^5
	LP353	0	54,750	<10/5 × 10^5
	LP362	0	101,100	>10/1.25 × 10^5
	LP366	0	28,125	>10/5 × 10^5
	LP377	0	290,400	>10/6.25 × 10^4
	LP381	0	52,200	>10/2.5 × 10^5
	LP391	0	99,600	<10/5 × 10^5
	LP409	0	92,800	>10/5 × 10^5
	LP413	0	197,600	>10/6.25 × 10^4
	LP418	0	93,600	<10/5 × 10^5
	LP424	0	52,800	>10/2.5 × 10^5
	LP426	0	213,600	>10/6.25 × 10^4
	LP432	0	51,300	>10/5 × 10^5
	LP434	0	194,400	>10/6.25 × 10^4
	LP435	0	67,950	>10/2.5 × 10^5
	LP454	0	25,425	<10/5 × 10^5
	LP456	0	40,000	>10/5 × 10^5
	LP462	0	20,700	<10/5 × 10^5
	LP469	0	90,000	>10/2.5 × 10^5
	LP472	0	80,800	<10/5 × 10^5
	LP475	0	155,700	>10/6.25 × 10^4

[a] SARS-CoV-2 Spike representing Wuhan-Hu-1 strain (WH1-S)

[b] Spot-formuing units (SFUs) were aggregated from 3 replicate wells seeded with 5 × 10^5 PBMC[s]

[c] SFU values were extrapolated to 1.5 × 10^6 PBMC

[d] Recombinant hemagglutinin (rHA) representing A/California/04/09 (H1N1) strain

[e] Lowest PBMC input yielding >10 SFU/well is denoted

Table S3: Post-COVID era donors possess variable frequencies of SARS-CoV-2 (WH1)-S-specific IgG^{+} B$_{mem}$

Post-COVID		WH1-S (FL)[a]	Pan IgG[a]
2023	LP714	173	45,760
	LP719	475	9310
	LP722	1259	19,140
	LP738	108	12,667
	LP741	24	23,840
	LP756	163	28,107
	LP757	520	52,960
	LP760	1291	32,120
	LP761	509	25,973
2024	LP803	848	31,720
	LP806	187	19,680
	LP807	768	27,264
	LP811	389	14,187
	LP815	188	26,080
	LP819	203	37,120
	LP821	3509	47,360
	LP822	397	24,427
	LP823	635	36,360
2025	LP826	723	20,080
	LP828	496	21,344
	LP829	373	17,840
	LP830	619	16,373
	LP831	573	32,760
	LP832	210	10,192
	LP833	367	20,380
	LP834	504	26,960
	LP836	168	28,560

[a]Spot-forming units (SFU) values were extrapoloated to 2×10^5 PBMCs

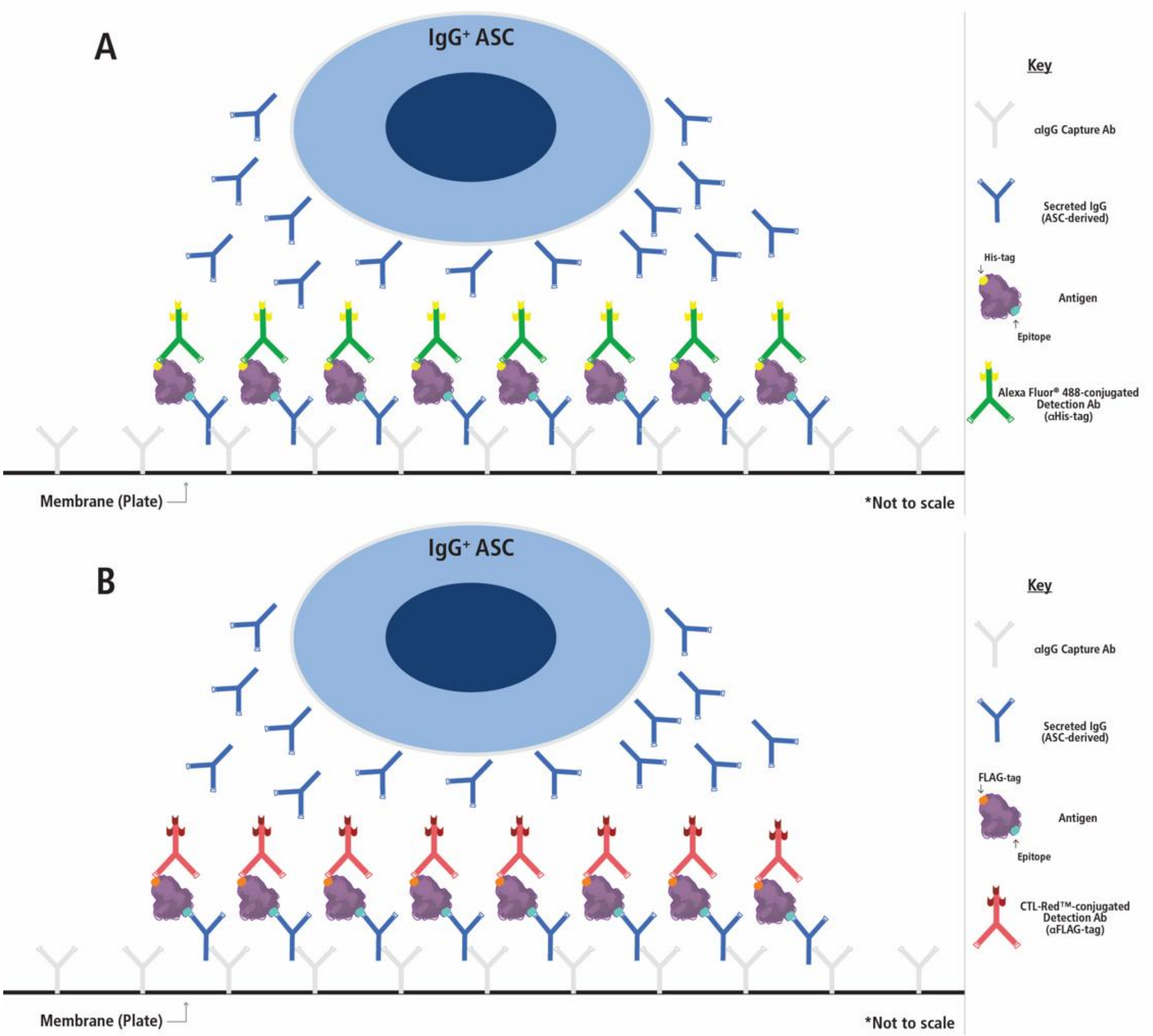

Figure S2: Illustration of single-color fluorescent, antigen-specific inverted B cell ImmunoSpot® test principle. In a single-color, fluorescent inverted ImmunoSpot® assay the membrane is coated with a pan anti-Ig class-specific (in this example IgG) capture antibody that will bind ASC-secreted IgG with high affinity irrespective of antigen specificity. Next, after removal of cells and washing of the assay plate(s), His-tagged (depicted in panel A) or FLAG-tagged (depicted in panel B) antigen probes are added at a sufficiently high concentration to ensure that they are retained by antigen-specific secretory footprints (IgG^+ in this example) possessing a spectrum of functional affinities. Antigen-specific secretory footprints that captured affinity-tagged antigen probes are subsequently revealed using Alexa Fluor® 488 conjugated anti-His (depicted in green, panel A) or CTL-Red™ conjugated anti-FLAG (depicted in red, panel B) detection antibodies. Individual spot-forming units (SFUs) can be quantified via image analysis following selective excitation and measurement of the resulting fluorescence using suitable instrumentation.

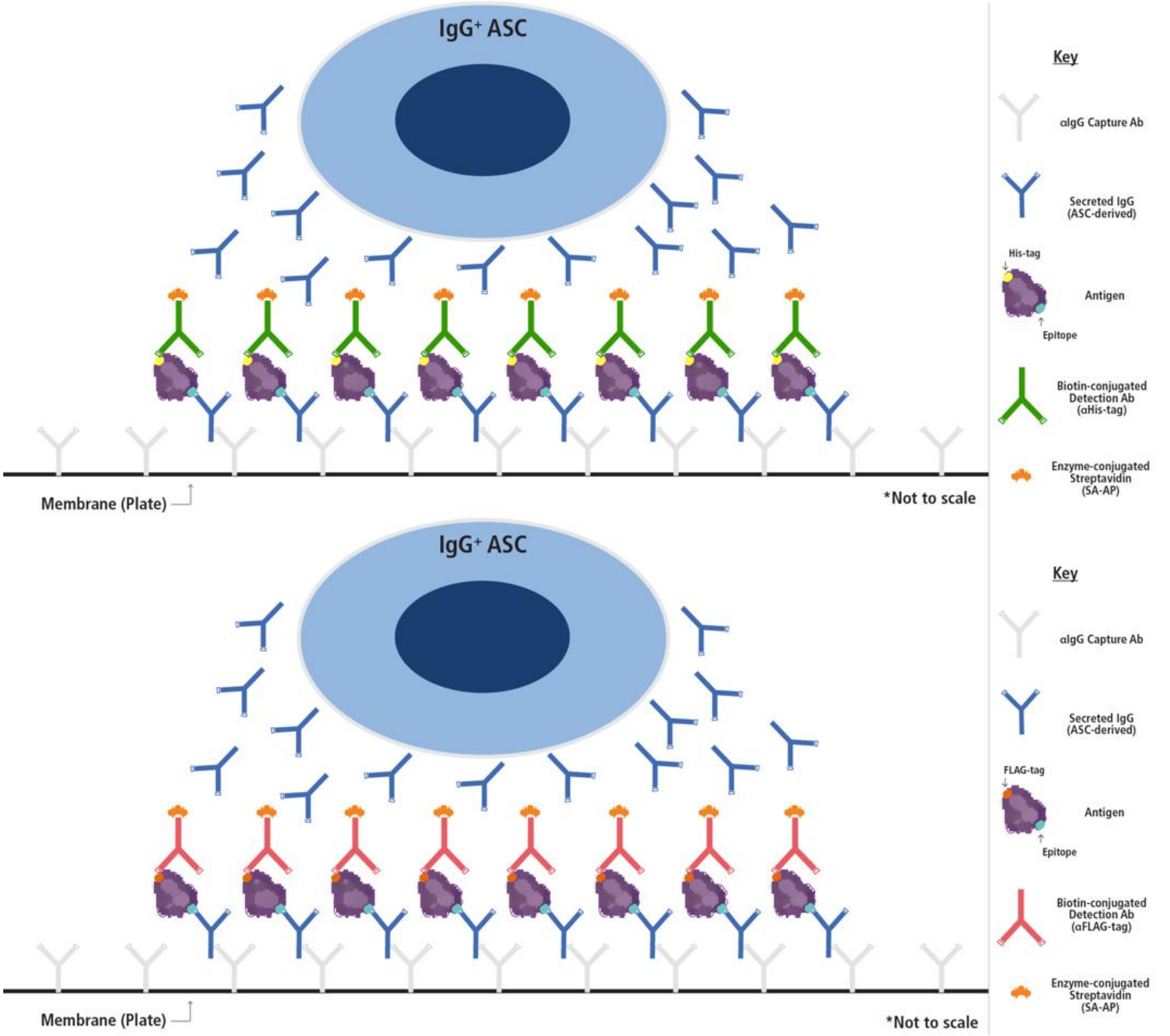

Figure S3: Illustration of single-color enzymatic, antigen-specific inverted B cell ImmunoSpot® test principle. In a single-color, enzymatic inverted ImmunoSpot® assay the membrane is coated with a pan anti-Ig class-specific (in this example IgG) capture antibody that will bind ASC-secreted IgG with high affinity irrespective of antigen specificity. Next, after removal of cells and washing of the assay plate(s), His-tagged (depicted in panel A) or FLAG-tagged (depicted in panel B) antigen probes are added at a sufficiently high concentration to ensure that they are retained by antigen-specific secretory footprints (IgG^{+} in this example) possessing a spectrum of functional affinities. Antigen-specific secretory footprints that captured affinity-tagged antigen probes are subsequently revealed using biotinylated anti-His (depicted in green, panel A) or anti-FLAG (depicted in red, panel B) detection antibodies, followed by addition of alkaline phosphatase-conjugated streptavidin (SA-AP). Individual spot-forming units (SFUs) are then visualized via deposition of a precipitating substrate and can be quantified via image analysis using suitable instrumentation.

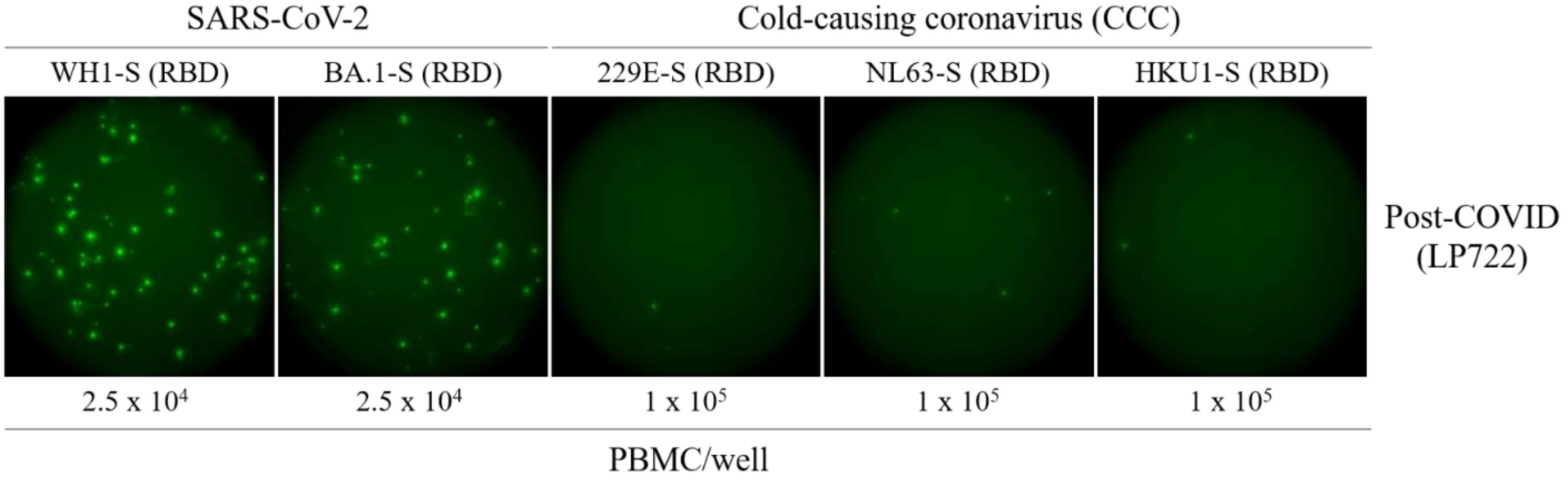

Figure S4: No evidence for B_{mem}-derived IgG^+ ASC cross-reactivity between the receptor binding domain (RBD) of cold-causing coronaviruses (CCCs) and SARS-CoV-2. Following five days of polyclonal stimulation to convert resting B_{mem} into ASCs, PBMCs from a post-COVID era donor (LP722, refer to Table S1) were plated at the indicated cell inputs into test wells previously coated with IgG-specific capture antibody and antigen-reactive secretory footprints were revealed using His-tagged probes representing the RBD expressed by different CCCs or SARS-CoV-2 strains (refer to Figure S2A for an illustration of the assay principle). Representative well images with contrast and brightness enhancements to aid in their visualization are shown. Notably, despite detecting elevated frequencies of SARS-CoV-2 RBD-reactive IgG^+ SFUs using either the ancestral (WH1) or variant (BA.1) RBD probes, few IgG^+ SFUs were revealed using RBD probes representing three different CCC strains despite plating four times the number of PBMCs in these assay

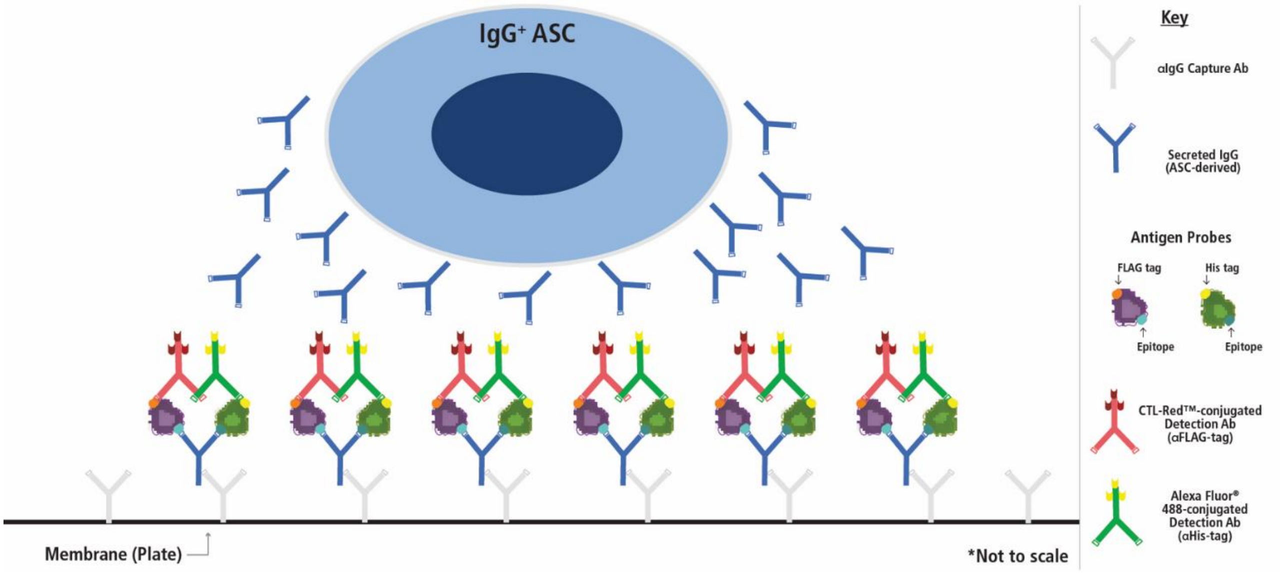

Figure S5: Illustration of dual-label fluorescent, antigen-specific inverted B cell ImmunoSpot® test principle. In an inverted ImmunoSpot assay, the membrane is coated with a pan anti-Ig class-specific (in this example IgG) capture antibody that will bind ASC-secreted IgG with high affinity irrespective of antigen specificity. Next, after removal of cells and washing of the assay plate(s), affinity-tagged (in this example His and FLAG) antigens are added at a sufficiently high concentration such that they will be retained by antigen-specific secretory footprints (IgG^+ in this example) possessing a spectrum of functional affinities. Antigen-specific secretory footprints that captured one or both affinity-tagged antigen probes are subsequently revealed using Alexa Fluor® 488 conjugated anti-His (depicted in green) and/or CTL-Red™ conjugated anti-FLAG (depicted in red) detection antibodies. Individual spot-forming units (SFUs) can be quantified via image analysis following selective excitation and measurement of the resulting fluorescence using suitable instrumentation. Notably, this method permits identification of strain-specific or cross-reactive ASCs at single-cell resolution based on whether one or both affinity-tagged antigens localize to an individual secretory footprint. In the schematic example shown, the ASC secreted IgG with specificity for both affinity-tagged antigens; hence, it would be classified as functionally cross-reactive.

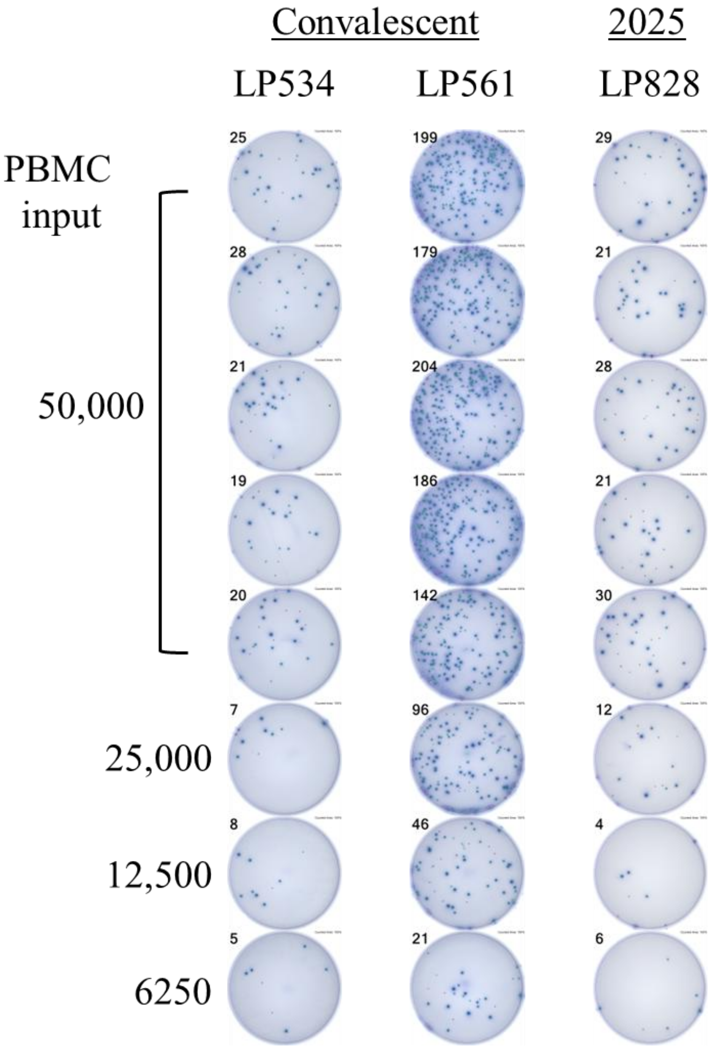

Figure S6: Testing strategy for determining donor-specific "Goldilocks" cell input for dual-label inverted ImmunoSpot® assays. Following five days of polyclonal stimulation to convert resting B_{mem} into ASCs, PBMCs were plated at the indicated cell inputs into test wells previously coated with IgG-specific capture antibody and antigen-reactive secretory footprints were revealed using His-tagged probe representing the ancestral SARS-CoV-2 strain (Wuhan-Hu-1, WH1-S (RBD)-His). Counted well images depicting the assay results obtained for the three indicated PBMC samples. Both LP534 and LP561 belong to the convalescent cohort and originate from donors with PCR-verified SARS-CoV-2 infections prior to availability of COVID-19 mRNA vaccines. LP828 was collected in the post-COVID era (2025) and has an unknown history of prior SARS-CoV-2 infection and/or COVID-19 vaccination. Based on these results, PBMCs from both LP534 and LP828 will be plated at 5×10^4 cells per well (the maximum cell input to ensure optimal assay performance and single-cell resolution) in follow-up dual-label inverted FluoroSpot assays. In contrast, owing to the elevated frequency of WH1-S (RBD)-reactive IgG^+ SFU, the LP561 sample will be plated at 1×10^4 cells per well to avoid SFU crowding and elevated membrane staining.

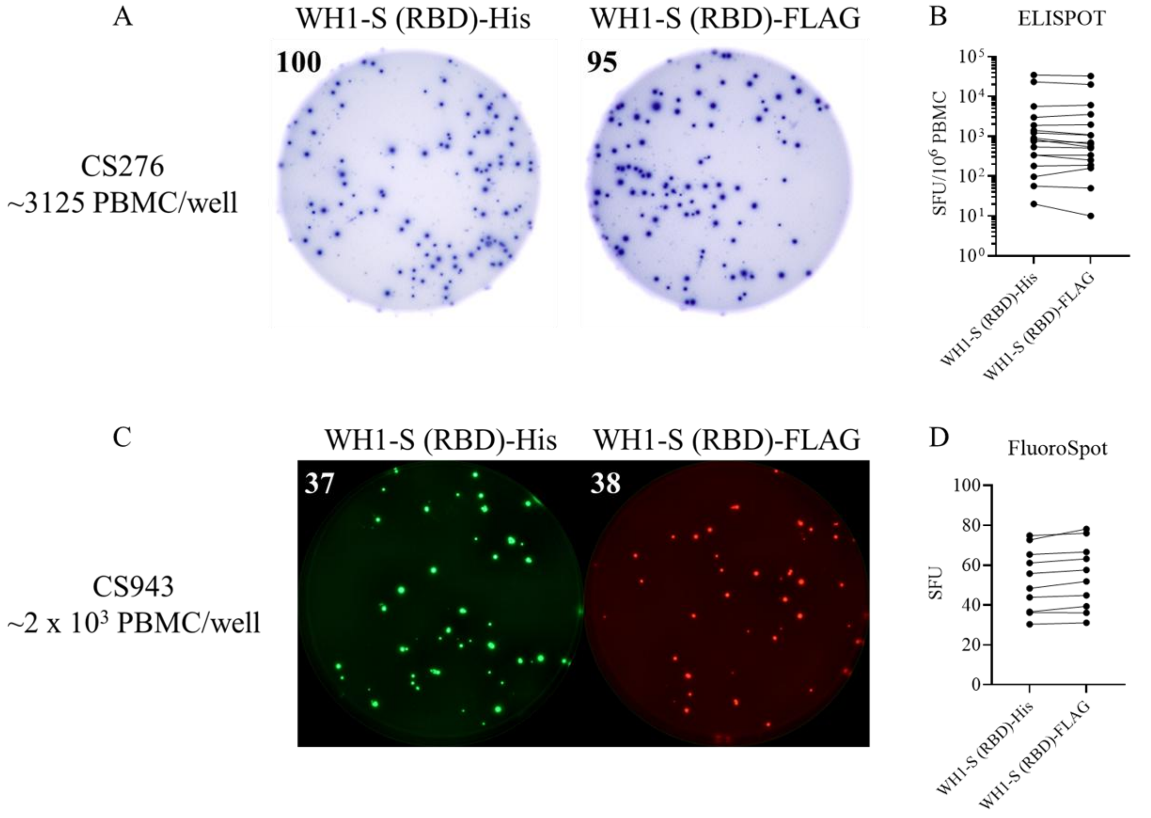

Figure S7: Single-color enzymatic or fluorescent inverted ImmunoSpot® assays provide comparable sensitivities for detection of WH1-S (RBD)-specific IgG$^+$ B$_{mem}$ irrespective of the affinity tag. A) Representative images depicting the detection of SARS-CoV-2 WH1-S (RBD)-specific B$_{mem}$-derived IgG$^+$ ASCs in single-color enzymatic (SCE) inverted ImmunoSpot® assays. The donor, PBMC input and machine-assisted count of individual spot-forming units (SFUs) are specified. Refer to Figure S3 for an illustration of the SCE inverted ImmunoSpot® assay principle. B) Paired frequencies of WH1-S (RBD)-specific B$_{mem}$-derived IgG$^+$ ASCs detected in SCE inverted ImmunoSpot® assay using a singlet serial dilution approach and either anti-His or anti-FLAG detection reagents. C) Representative well images, with contrast and brightness enhancements applied to aid in their visualization, depicting the detection of WH1-S (RBD)-specific IgG$^+$ B$_{mem}$-derived IgG$^+$ ASCs in single-color fluorescent (SCF) inverted ImmunoSpot® assays. The donor, PBMC input and machine-assisted count of individual SFUs are specified. Refer to Figure S2 for an illustration of the SCF inverted ImmunoSpot® assay principle. D) Mean SFU count from nine replicate wells seeded with donor-specific PBMC inputs, previously defined to yield SFUs in the Goldilocks range of 30-80 SFUs/well, are shown from SCF assays detected with either the His-tagged or FLAG-tagged WH1-S (RBD) antigen probes and the corresponding detection reagents.

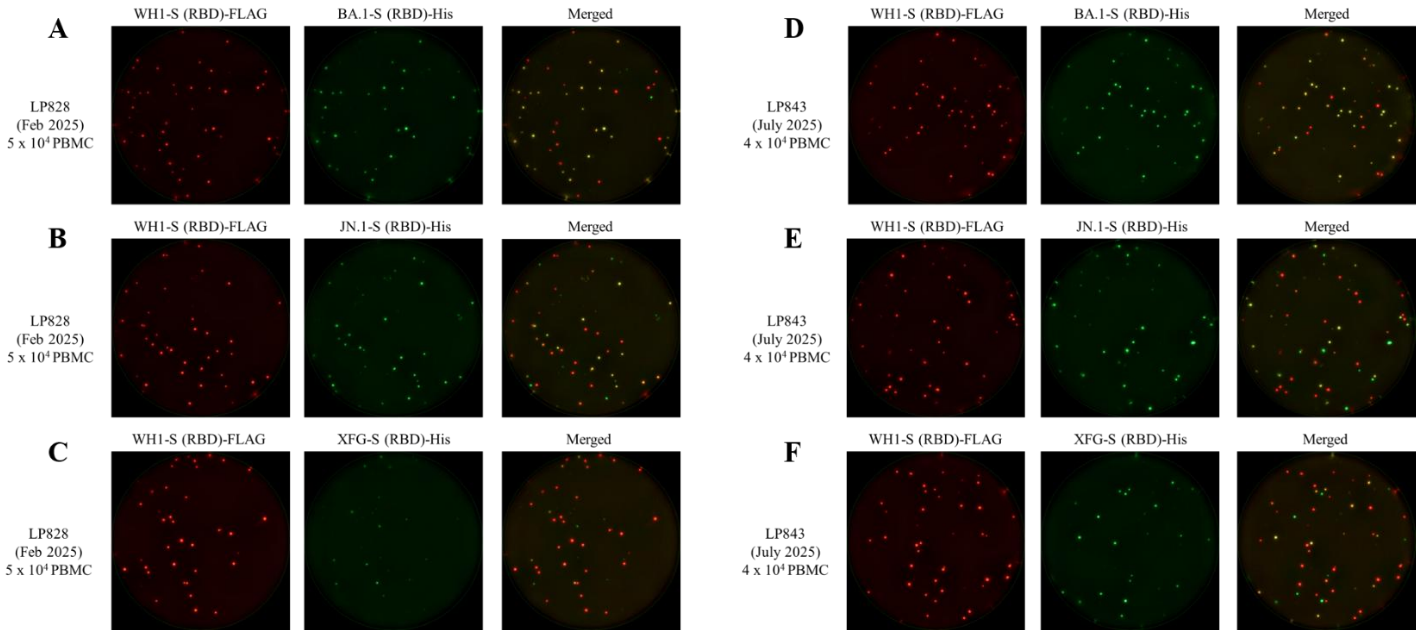

Figure S8: Dual-label inverted FluoroSpot permits distinction between strain-specific and cross-reactive ASCs. A-C) Representative well images depicting dual-label inverted FluoroSpot assay results obtained when testing polyclonally stimulated PBMC from a donor collected in February 2025 (LP828, refer to Table S1). In panel A, well images depicting detection of individual spot-forming units (SFUs) using WH1-S (RBD)-FLAG or BA.1-S (RBD)-His RBD antigen representing a future SARS-CoV-2 variant. Additionally, the well images were merged (virtual overlay of red and green fluorescence detection planes) using the ImmunoSpot® software to visualize dual-labeling of SFUs. Likewise, the same donor was also tested using (panel B) JN.1-S (RBD)-His or (panel C) XFG-S (RBD)-His antigens representing SARS-CoV-2 variants in combination with the WH1-S (RBD)-FLAG probe. D-F) Representative well images depicting results obtained when testing PBMC from a donor collected in July 2025 (LP843, refer to Supplementary Table 1). In panel D, well images depicting detection of SFUs using WH1-S (RBD)-FLAG or BA.1-S (RBD)-His RBD antigen probes, along with a virtual overlay of the color planes. The same donor was also tested using (panel E) JN.1-S (RBD)-His or (panel F) XFG-S (RBD)-His antigens representing SARS-CoV-2 variants in combination with the WH1-S (RBD)-FLAG probe. Note: images were contrast enhanced and adjusted for brightness to aid visualization.

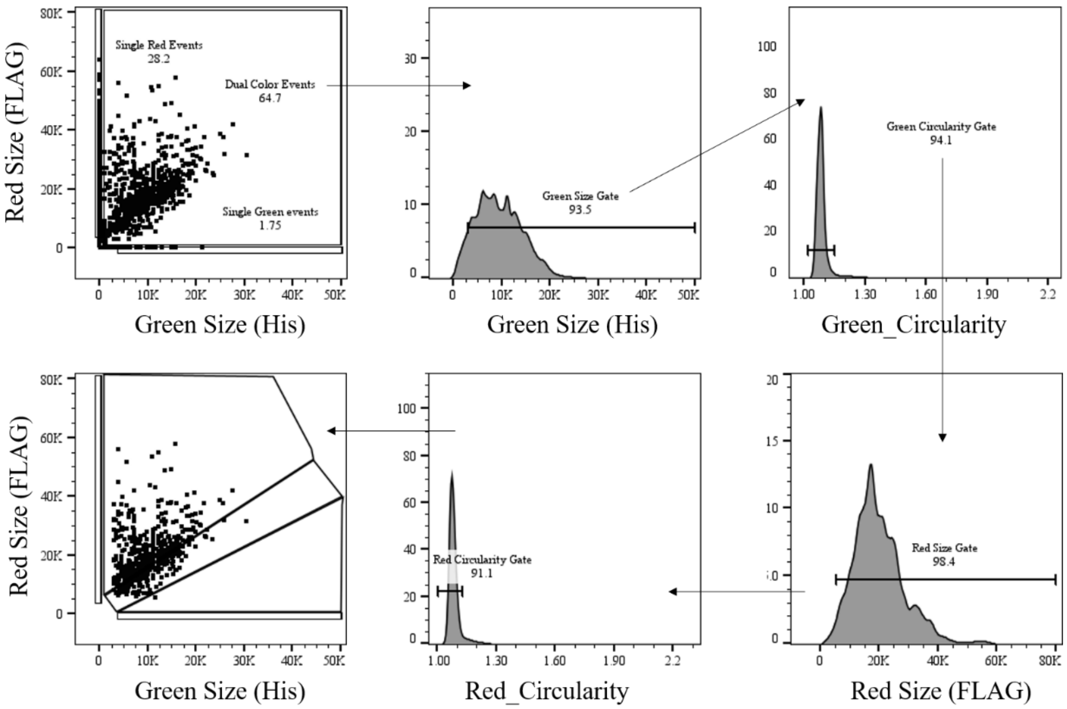

Figure S9: Extended gating scheme for analysis of inverted FluoroSpot data. Gating scheme for identification and segregation of dual-labeled spot-forming units (SFUs) is depicted. The representative plots and arrows denote the analysis workflow for the SFUs detected when testing PBMCs from CS134 (a donor with PCR-verified SARS-CoV-2 infection in early 2022) using the combination of WH1-S (RBD)-FLAG and BA.1-S (RBD)-His probes. As the first step, SFUs from merged wells are segregated into single-positive (SP) or double-positive (DP) events. Next, the events (DP in this example) are further gated to exclude secretory footprints below a minimal size and those possessing an elevated circularity value (denoting deviation from a perfect circle and an irregular footprint outline). DP events must satisfy all these criteria for inclusion, whereas SP events were only restricted on the green or red minimal size and circularity metrics, respectively. The final plot depicts the segregation of dual-labeled SFUs into the three subcategories: those with proportionally equivalent secretory footprint sizes and those exhibiting larger footprints representing a particular RBD probe.

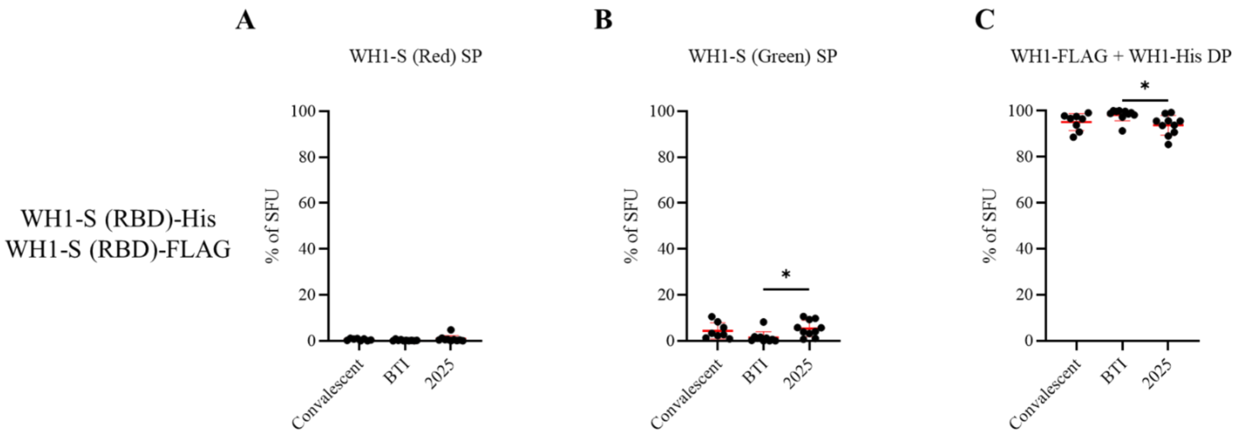

Figure S10: Frequency of single-positive (SP) and double-positive (DP) SARS-CoV-2-specific SFU revealed using WH1-S (RBD)-FLAG and WH1-S (RBD) His antigen probes. Polyclonally stimulated PBMC originating from donors that (*i*) recovered from PCR-verified SARS-CoV-2 infections in 2020 (Convalescent) prior to availability of COVID-19 vaccines (n=8), (*ii*) recovered from PCR-verified SARS-CoV-2 infection in early 2022 (breakthrough infection, BTI) (n=10), or (*iii*) collected in 2025 without defined histories of past infection(s) or vaccination(s) (n=10) were tested in dual label inverted ImmunoSpot® assays (refer to Section ?). A-C) Frequency of SP or DP secretory footprints detected using the combination of WH1-S (RBD)-FLAG and WH1-S (RBD)-His antigen probes. Statistical significance (* $p<0.05$) of difference between donor cohorts were determined using Welch's analysis of variance (ANOVA) test with Dunnett's T3 post-hoc correction for multiple comparisons.

Table S4: Cumulative number of single-positive (SP) and double-positive (DP) SFUs detected in dual-label inverted ImmunoSpot® assays

		WH1 (Red) + WH1 (Green)			WH1 (Red) + BA.1 (Green)			WH1 (Red) + JN.1 (Green)			WH1 (Red) + XFG (Green)		
		Single Red	Single Green	Dual Labeled	Single Red	Single Green	Dual Labeled	Single Red	Single Green	Dual Labeled	Single Red	Single Green	Dual Labeled
Convalescent	LP526	0	30	866	578	28	225	737	30	127	768	22	109
	LP534	2	29	467	306	44	242	374	34	156	368	36	105
	LP553	8	77	651	403	76	337	584	68	162	571	64	172
	LP560	6	8	612	336	11	356	418	9	192	483	11	187
	LP561	0	6	675	434	6	272	553	4	157	537	2	136
	LP566	2	22	930	416	20	515	647	17	292	714	15	275
	LP568	5	44	484	284	78	298	482	48	127	444	52	114
	LP569	8	27	974	651	27	436	900	25	287	891	40	295
BTI[a]	CS015	7	14	746	441	31	399	560	37	167	669	30	148
	CS134	2	13	805	233	16	518	634	16	174	650	11	182
	CS178	2	5	861	424	26	419	648	23	221	700	14	205
	CS211	2	32	356	157	24	232	283	37	99	208	32	86
	CS276	0	2	180	135	1	66	212	2	26	231	0	14
	CS813	0	0	514	209	3	237	422	2	110	431	1	85
	CSF814	1	0	534	264	1	252	509	1	76	475	0	66
	CS900	1	6	511	139	3	355	343	9	187	371	6	148
	CS943	0	0	897	403	6	468	683	2	187	731	4	184
	CS946	0	1	405	229	0	231	329	0	108	265	5	58
2025	LP826	1	8	802	222	48	599	652	17	246	744	17	210
	LP828	3	29	690	252	33	487	305	47	409	359	46	257
	LP837	0	1	147	45	11	102	97	18	58	120	5	42
	LP840	3	8	236	120	16	153	217	13	91	210	15	87
	LP842	20	41	356	63	54	337	227	47	210	234	43	157
	LP843	4	29	707	282	68	529	388	143	394	467	197	357
	LP853	3	34	561	92	81	460	249	146	313	342	106	291
	LP854	3	29	469	98	82	392	255	52	219	311	45	182
	LP860	3	77	647	103	152	601	275	217	410	300	179	387
	LP863	0	82	801	232	90	693	536	91	347	592	101	276

[a]Breakthrough infection

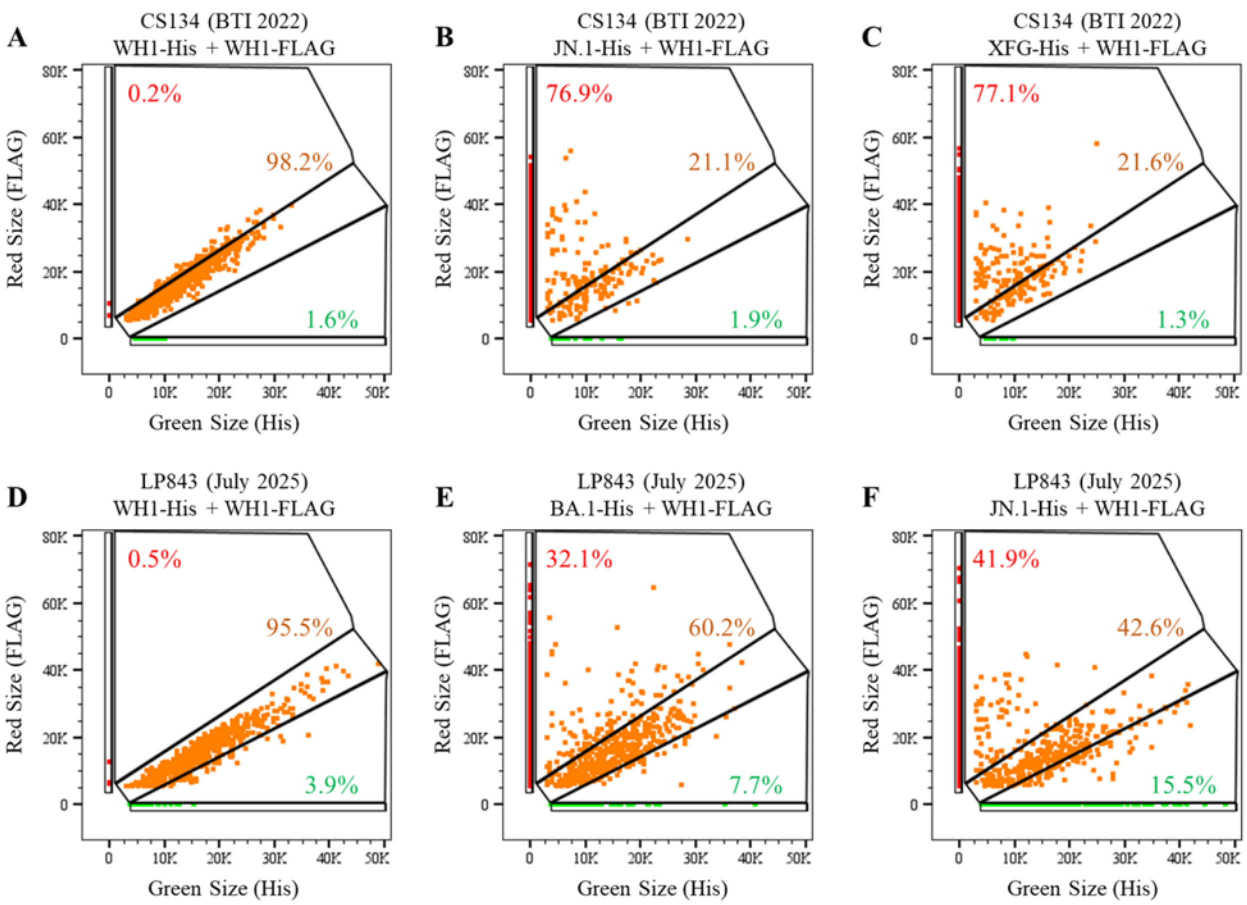

Figure S11: High content data analysis of dual-label inverted FluoroSpot assay results. A-C) Flow cytometry standard (FCS) plots depicting the segregation of spot-forming units (SFUs) generated by CS134 (collected in 2022) using WH1-S (RBD)-FLAG antigen probe in combination with WH1-S (RBD)-His (panel A), JN.1-S (RBD)-His (panel B), or XFG-S (RBD) His (panel C) probes. D-F) FCS plots depicting the segregation of SFUs generated by LP843 (collected in July 2025) using WH1-S (RBD)-FLAG antigen probe in combination with WH1-S (RBD)-His (panel D), BA.1-S (RBD)-His (panel E), or JN.1-S (RBD) His probes (panel F). Notably, nearly all SFUs for both CS134 and LP843 were dual-labeled using antigenically matched WH1-S (RBD)-FLAG and WH1-S (RBD)-His probes. In contrast, an increased number of WH1-S single-positive (SP) SFUs was observed for both CS134 and LP843 using RBD antigen probes representing SARS-CoV-2 variants. Moreover, an increase in the number of SARS-CoV-2 variant RBD SP SFUs was also observed when testing LP843 (see panels E and F).

Table S5: Cumulative number of double-positive (DP) SFUs with distinct secretory footprints sizes in dual-label inverted ImmunoSpot® assays.

		WH1 (Red) + WH1 (Green)				WH1 (Red) + BA.1 (Green)				WH1 (Red) + JN.1 (Green)				WH1 (Red) + XFG (Green)			
		Dual Labeled	Larger Red	Larger Green	Equal (Red/Green)	Dual Labeled	Larger Red	Larger Green	Equal (Red/Green)	Dual Labeled	Larger Red	Larger Green	Equal (Red/Green)	Dual Labeled	Larger Red	Larger Green	Equal (Red/Green)
Convalescent	LP526	866	49	1	816	225	124	4	97	127	39	15	73	109	54	7	48
	LP534	467	0	96	371	242	25	25	192	156	105	15	36	105	49	10	46
	LP553	651	0	330	321	337	57	41	239	162	42	29	91	172	60	32	80
	LP560	612	29	1	582	356	106	0	250	192	69	5	118	187	60	1	126
	LP561	675	19	0	656	272	53	3	216	157	45	5	107	136	46	0	90
	LP566	930	6	2	922	515	39	78	398	292	128	17	147	275	115	9	151
	LP568	484	0	84	400	298	48	18	232	127	33	10	84	114	28	10	76
	LP569	974	0	220	754	436	84	32	320	287	110	37	140	295	127	48	120
BTI[a]	CS015	746	49	4	693	399	168	18	213	167	105	5	57	148	101	6	41
	CS134	805	44	1	760	518	328	1	189	174	70	5	99	182	84	0	98
	CS178	861	94	0	767	419	198	6	215	221	69	22	130	205	100	12	93
	CS211	356	0	50	306	232	40	0	192	99	24	5	70	86	25	1	60
	CS276	180	0	0	180	66	9	0	57	26	7	0	19	14	5	0	9
	CS813	514	104	0	410	237	111	5	121	110	31	2	77	85	48	1	36
	CSF814	534	285	0	249	252	195	0	57	76	15	1	60	66	30	4	32
	CS900	511	12	0	499	355	126	23	206	187	66	0	121	148	54	0	94
	CS943	897	5	1	891	468	145	0	323	187	50	2	135	184	54	6	124
	CS946	405	0	10	395	231	85	0	146	108	22	1	85	58	23	0	35
2025	LP826	802	0	11	791	599	91	25	483	246	56	18	172	210	54	0	156
	LP828	690	1	9	680	487	34	9	444	409	182	22	205	257	116	3	138
	LP837	147	0	0	147	102	5	1	96	58	10	3	45	42	4	0	38
	LP840	236	0	6	230	153	49	1	103	91	26	0	65	87	18	1	68
	LP842	356	0	79	277	337	60	29	248	210	31	24	155	157	17	30	110
	LP843	707	0	56	651	529	91	42	396	394	58	93	243	357	131	3	223
	LP853	561	0	37	524	460	164	6	290	313	113	19	181	291	94	30	167
	LP854	469	0	112	357	392	77	54	261	219	32	47	140	182	29	21	132
	LP860	647	0	114	533	601	120	8	473	410	72	12	326	387	157	11	219
	LP863	801	0	219	582	693	213	19	461	347	79	84	184	276	46	60	170
